# Supplementary material for: Rapid Characterization of hERG Channel Kinetics I: Using an Automated High-Throughput System
Source: Biophys J. 2019 Jul 25;117(12):2438–54. doi: 10.1016/j.bpj.2019.07.029 (PMC6990155; doi:10.1016/j.bpj.2019.07.029)
Supplement: Document S2. Article plus Supporting Material [file mmc2.pdf]

Biophysical Journal
Article

Biophysical Society

Rapid Characterization of hERG Channel Kinetics I: Using an Automated High-Throughput System

Chon Lok Lei,[1] Michael Clerx,[1] David J. Gavaghan,[1] Liudmila Polonchuk,[2] Gary R. Mirams,[3,*] and Ken Wang[2]
[1]Computational Biology, Department of Computer Science, University of Oxford, Oxford, United Kingdom; [2]Pharma Research and Early Development, Innovation Center Basel, F. Hoffmann-La Roche Ltd., Basel, Switzerland; and [3]Centre for Mathematical Medicine and Biology, School of Mathematical Sciences, University of Nottingham, Nottingham, United Kingdom

ABSTRACT Predicting how pharmaceuticals may affect heart rhythm is a crucial step in drug development and requires a deep understanding of a compound's action on ion channels. In vitro hERG channel current recordings are an important step in evaluating the proarrhythmic potential of small molecules and are now routinely performed using automated high-throughput patch-clamp platforms. These machines can execute traditional voltage-clamp protocols aimed at specific gating processes, but the array of protocols needed to fully characterize a current is typically too long to be applied in a single cell. Shorter high-information protocols have recently been introduced that have this capability, but they are not typically compatible with high-throughput platforms. We present a new 15 second protocol to characterize hERG (Kv11.1) kinetics, suitable for both manual and high-throughput systems. We demonstrate its use on the Nanion SyncroPatch 384PE, a 384-well automated patch-clamp platform, by applying it to Chinese hamster ovary cells stably expressing hERG1a. From these recordings, we construct 124 cell-specific variants/parameterizations of a hERG model at 25°C. A further eight independent protocols are run in each cell and are used to validate the model predictions. We then combine the experimental recordings using a hierarchical Bayesian model, which we use to quantify the uncertainty in the model parameters, and their variability from cell-to-cell; we use this model to suggest reasons for the variability. This study demonstrates a robust method to measure and quantify uncertainty and shows that it is possible and practical to use high-throughput systems to capture full hERG channel kinetics quantitatively and rapidly.

SIGNIFICANCE We present a method for high-throughput characterization of hERG potassium channel kinetics via fitting a mathematical model to results of over 100 single-cell patch-clamp measurements collected simultaneously on an automated voltage-clamp platform. The automated patch-clamp data are used to parameterize a mathematical ion channel model fully, opening a new era of automated and rapid development of mathematical models from quick, cheap, and reliable experiments. The method also allows ample data for independent validation of the models and enables us to study experimental variability and its origins. The method can be applied to characterize different conditions, e.g., temperatures (see Part II), mutations, or the action of pharmaceuticals, and could be adapted to study many other currents.

INTRODUCTION

The *human Ether-à-go-go-Related Gene* (*hERG*) is of great importance in cardiac electrophysiology and safety pharmacology. *hERG* encodes the pore-forming α subunit of the ion channel Kv11.1, which conducts the rapid delayed rectifier potassium current, I_{Kr} (1). Reduction of I_{Kr} by pharmaceutical compounds or mutations can prolong the ventricular action potential (2), can increase the QT interval on the body-surface electrocardiogram, and is associated with elevated risk of Torsade de Pointes (3). Current pharmaceutical regulatory guidelines require the evaluation of effects on the hERG channel as part of preclinical drug development (4).

High-throughput automated patch-clamp screening for ion current inhibition by pharmaceutical compounds has been widely used to inform proarrhythmic safety in early drug discovery. Inhibition data from multiple ion channels can be integrated together using a mechanistically detailed in silico electrophysiology model to predict proarrhythmic risk (5). Such a strategy, combining high-throughput in vitro and in silico approaches, is being advocated by a Food and Drug Administration-led initiative, the Comprehensive in vitro Proarrhythmia Assay (6), as a core pillar

Submitted April 15, 2019, and accepted for publication July 17, 2019.
*Correspondence: gary.mirams@nottingham.ac.uk
Editor: Zhilin Qu.
https://doi.org/10.1016/j.bpj.2019.07.029
© 2019 Biophysical Society.
This is an open access article under the CC BY license (http://creativecommons.org/licenses/by/4.0/).

of future proarrhythmic safety assessment. High-throughput automated patch-clamp has also been used to characterize the kinetics of a large number of *KCNQ1* mutants that were previously variants of unknown significance (7).

Mathematical modeling of ion channel kinetics provides a quantitative summary of our current understanding, and can serve as a powerful predictive tool. The parameters in ion current models can be biophysically and physiologically meaningful and are therefore of interest in their own right. Parameterization (or calibration) of mathematical models is a concise way to characterize ion current kinetics and can also be used to quantify variability between experiments (8). A wide range of models have been proposed to describe I_{Kr}, with varying levels of biophysical detail and numbers of parameters (see Beattie et al. (9), Appendix A)). Until we have a full and clear understanding of the underlying mechanisms, simple models that capture the most relevant characteristics with a small number of parameters may be preferred.

Voltage-clamp experiments are a common source of data for calibrating ion channel models. The first models of ionic currents were proposed by Hodgkin and Huxley (10), who used stepwise voltage protocols to isolate and measure different aspects of ionic currents (e.g., time constants and voltage-dependent steady states). Following in their footsteps, many voltage step protocols have been designed to highlight particular current kinetics. Typically, these protocols involve long sections, during which the channels are brought into a particular steady state before a brief interval, during which a current is measured and then summarized using either a peak current or by fitting an exponential curve and deriving a time constant. By design, these protocols focus on a single aspect of an ion current, so several such protocols are needed to parameterize a model fully. For the hERG channel, for example, in (11), examining voltage dependence of hERG activation lasted at least 10 min before examining deactivation, inactivation, and recovery from inactivation; hence, these protocols are typically too long for a single-cell recording. More recently, simulation experiments have shown that condensed voltage-clamp protocols can be used to provide the required information in a much shorter time (12,13). A study by Beattie et al. (9) demonstrated in vitro that sinusoidal protocols can be used to rapidly (8 s) characterize hERG kinetics on a manual patch-clamp setup. Because of hardware limitations, some automated high-throughput systems can only perform square wave or ramp voltage-clamp protocols. Here, we extend the approach of Beattie et al. (9) to make it applicable to such automated high-throughput patch-clamp systems.

Efforts have been made to address the variability observed in measurements of the hERG channel (14). However, the variability of baseline hERG characteristics remains incompletely understood. Understanding and quantifying this variability, whether it is due to cell-to-cell variability (also known as "extrinsic variability" or "population variability") or to observational errors/uncertainties, is crucial in establishing the credibility and applicability of model predictions (15). Quantifying the variability in hERG channel kinetics requires a large number of high-quality patch-clamp measurements and an appropriate statistical framework. The duration of a standard combination of protocols makes it difficult to use them to fully characterize the current in a single cell, so that reaching the required number of cells for a thorough statistical analysis would be a very difficult and time-consuming task.

We present a new approach to overcome this problem by using a novel protocol and a high-throughput system to rapidly record many cells' kinetics in parallel. Using these methods, we construct 124 cell-specific parameterizations of a hERG model and validate all of our model predictions against a set of independent protocols that have not been used in training or fitting the model. To ensure the stability and reproducibility of our results within the same cells, we repeat all of our measurements twice. We employ a hierarchical Bayesian framework (a multilevel statistical modeling technique) to describe the variability of hERG channel conductance and kinetics between cells and to infer the covariance between the model parameters across different cells. This study greatly increases the utility of automated high-throughput systems and provides robust tools for the uncertainty quantification that comprise an essential component of an in silico assay.

MATERIALS AND METHODS

We began our work with a synthetic data study to inform the experimental design of the voltage protocols, and applied inference techniques to assess the amount of information such protocols can provide. The motivation and rationale of our newly designed protocol are discussed in the Experimental Methods. Experiments using this new protocol were performed on the Nanion SyncroPatch 384PE platform (Nanion Technologies, Munich, Germany) with a temperature control unit. We then applied global optimization, Markov chain Monte Carlo (MCMC), and hierarchical Bayesian techniques to recover parameters for a mathematical ion current model for each individual cell, as described below.

Mathematical model

We used a recently published hERG model by Beattie et al. (9), which has a Hodgkin and Huxley-style structure. This model structure has been widely used in many studies with slight modifications: the root of the model traces back to Zeng et al. (16), in which the same model structure was used but with the inactivation gate modeled as an instantaneous steady-state response. Later, in the ten Tusscher et al. (17) model, the same model structure was used, but extra parameters were introduced to make the time constant independent of the steady state. In the model that we use, the current, I_{Kr}, is modeled with a standard Ohmic expression,

$$I_{\mathrm{Kr}} = g_{\mathrm{Kr}} a r (V - E_K), \tag{1}$$

where g_{Kr} is the maximal conductance, a is a Hodgkin and Huxley (10) activation gate, and r is an inactivation gate. V is the transmembrane voltage and E_K is the reversal potential, also known as the Nernst potential. E_K was not inferred but was calculated directly using

$$E_K = \frac{RT}{zF} \ln\left(\frac{[\mathrm{K}^+]_\mathrm{o}}{[\mathrm{K}^+]_\mathrm{i}}\right), \quad (2)$$

where R is the ideal gas constant, T is the absolute temperature, F is the Faraday constant, and z is the valency of the ions (equal to 1 for K^+). $[K^+]_o$ and $[K^+]_i$ denote the extracellular and intracellular concentrations of K^+, respectively, which were determined by the experimental solutions as 4 and 110 mM, respectively. The model structure is shown in Fig. 1, where

$$\frac{\mathrm{d}a}{\mathrm{d}t} = \frac{a_\infty - a}{\tau_a}, \quad \frac{\mathrm{d}r}{\mathrm{d}t} = \frac{r_\infty - r}{\tau_r},$$
$$a_\infty = \frac{k_1}{k_1 + k_2}, \quad r_\infty = \frac{k_4}{k_3 + k_4},$$
$$\tau_a = \frac{1}{k_1 + k_2}, \quad \tau_r = \frac{1}{k_3 + k_4},$$

where

$$k_1 = p_1 \exp(p_2 V), \quad k_3 = p_5 \exp(p_6 V),$$
$$k_2 = p_3 \exp(-p_4 V), \quad k_4 = p_7 \exp(-p_8 V).$$

Our model consists of nine positive parameters $\boldsymbol{\theta} = \{g_{\mathrm{Kr}}, p_1, ..., p_8\}$, where the units of the parameters are $\{\mathrm{pS}, \mathrm{s}^{-1}, \mathrm{V}^{-1}, \mathrm{s}^{-1}, \mathrm{V}^{-1}, ...\}$. All model parameters must be inferred from the experimental data.

Simulations were run using Myokit (18), with tolerance settings for the CVODE solver (19) set to $abs\_tol = 10^{-8}$ and $rel\_tol = 10^{-10}$. All codes and data are freely available at https://github.com/CardiacModelling/hERGRapidCharacterisation.

Statistical model and parameter inference

To infer model parameters from experimentally observed data under a probabilistic and Bayesian framework, we specified a statistical model to relate the mathematical model and the observed experimental data:

$$I_{\mathrm{Kr}}^{\mathrm{data}} = I_{\mathrm{Kr}}^{\mathrm{model}} + \epsilon. \quad (3)$$

We assumed that noise arises from a normal distribution $\epsilon \sim \mathcal{N}(0, \sigma^2)$. This is equivalent to writing $I_{\mathrm{Kr}}^{\mathrm{data}} \sim \mathcal{N}(I_{\mathrm{Kr}}^{\mathrm{model}}, \sigma^2)$, which allows us to formulate the likelihood of observing the data $\mathbf{y} = \{y_k\}$ given parameters $\boldsymbol{\phi} = \ln(\boldsymbol{\theta})$ as

$$p(\mathbf{y}|\boldsymbol{\phi}, \sigma) = \frac{1}{\sqrt{2\pi\sigma^2}} \exp\left(-\sum_k \frac{(z_k(\boldsymbol{\phi}) - y_k)^2}{2\sigma^2}\right), \quad (4)$$

where $\mathbf{z} = \{z_k\}$ is the model simulation of $I_{\mathrm{Kr}}^{\mathrm{model}}$ given $\boldsymbol{\theta}$. We chose the parameter transformation $\boldsymbol{\phi} = \ln(\boldsymbol{\theta})$ to turn our positively constrained physical model parameters to be unconstrained optimization variables. Using Bayes' theorem, we can now write an equation for the likelihood of a parameter set given the observed data (the posterior) as

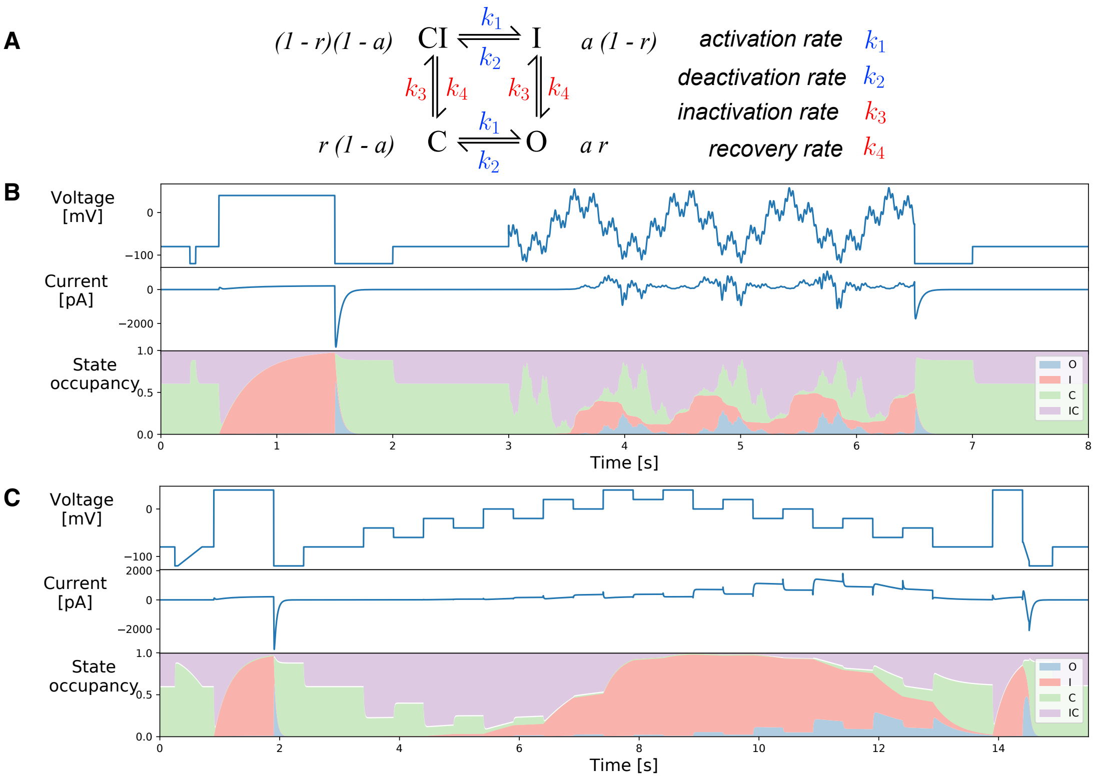

FIGURE 1 (*A*) The Hodgkin-Huxley model structure shown in equivalent Markov state diagram format. Four states are linked with arrows, with rate transitions described on the right. The probabilities of each state are given next to them in terms of the Hodgkin-Huxley gates a and r. (*B*) A manual patch-clamp protocol composed of an 8 s voltage-clamp protocol designed for rapid characterization of ion channel kinetics by Beattie et al. (9) is shown. (*C*) Our novel 15 s protocol, which we term the "staircase protocol," is shown, designed for any patch-clamp set-up, including high-throughput automated systems, which is similarly able to characterize the full kinetics of our hERG channel model. Both (*B*) and (*C*) show the voltage protocol (*top panel*), an example of the simulated current using the room temperature parameters from the work of Beattie et al. (9) cell #5 (*middle panel*), and the corresponding state occupancy (*bottom panel*). To see this figure in color, go online.

$$p(\boldsymbol{\phi}, \sigma \mid \mathbf{y}) = \frac{p(\boldsymbol{\phi})p(\mathbf{y} \mid \boldsymbol{\phi}, \sigma)}{p(\mathbf{y})}, \quad \propto p(\boldsymbol{\phi})p(\mathbf{y} \mid \boldsymbol{\phi}, \sigma), \tag{5}$$

with the prior

$$p(\boldsymbol{\phi}) \sim \mathcal{U}\left(\boldsymbol{\phi}^{\min}, \boldsymbol{\phi}^{\max}\right), \tag{6}$$

where $\mathcal{U}(\bullet)$ represents a uniform distribution.

Here, $\mathbf{y}$ was assumed to be the $I_{\mathrm{Kr}}^{\mathrm{data}}$ in Eq. 3 after leak correction and E-4031 subtraction have been applied as part of the data processing. We chose a uniform prior and expected our posterior to be dominated by the observed data. The details of the choice of $\boldsymbol{\phi}^{\min}$, $\boldsymbol{\phi}^{\max}$ are given in Supporting Materials and Methods, Section S6.2.2. Such a formulation extends our model parameters to $\{\boldsymbol{\theta}, \sigma\}$ to fully describe both the biophysical and statistical models.

We used a two-step approach to infer the model parameters. Firstly, we used a global optimization algorithm (20) to identify the parameters. Secondly, we utilized a Monte-Carlo-based sampling scheme to obtain the posterior distribution, using a population MCMC (21) algorithm with adaptive Metropolis (22) as the base sampler. The benefits of this approach are twofold. First, using a Bayesian framework allows us to incorporate prior knowledge. Second, we construct a probability (posterior) distribution to quantify uncertainty in the parameter set due to noise in the data. All inference and sampling were done via our open-source Python package, PINTS (23).

Hierarchical Bayesian model

We combined multiple experimental recordings using a multilevel modeling technique known as a hierarchical Bayesian model. Under this framework, we assume the vector of the transformed parameters ϕ for a particular cell follows a multivariate normal distribution that describes how these parameters are distributed between all cells, namely $\boldsymbol{\phi} \sim \mathcal{N}(\boldsymbol{\mu}, \boldsymbol{\Sigma})$. Given our choice of parameter transformation, this is equivalent to writing $\boldsymbol{\theta} \sim \mathrm{LogNormal}(\boldsymbol{\mu}, \boldsymbol{\Sigma})$, that is, the vector of parameters $\boldsymbol{\theta}$ for a particular cell follows a multivariate log-normal distribution. Then, we used the hierarchical Bayesian model to infer the mean vector $\boldsymbol{\mu}$ and covariance matrix $\boldsymbol{\Sigma}$ across cells and hence determined any correlation in model parameter sets between cells. The parameter dependency for this hierarchical Bayesian model is shown in Fig. S5.

The full hierarchical Bayesian likelihood $\mathcal{L}$ was specified as the product of 1) the probability of producing data $\mathbf{y}_j$ on each cell j given the parameter vector for each cell $\boldsymbol{\theta}_j$ and noise σ_j; 2) the probability of obtaining each individual well parameter set $\boldsymbol{\theta}_j$ from the "top-level" LogNormal distribution across wells defined by the hyperparameters; and 3) the priors—the prior of the hyperparameters (also known as the "hyperprior") and the prior of σ_j. That is,

$$\mathcal{L}\left(\boldsymbol{\mu}, \sum, \{\boldsymbol{\theta}_j, \sigma_j\}_{j=1}^{N_e} \middle| \{\mathbf{y}_j\}_{j=1}^{N_e}\right) \propto \prod_{j=1}^{N_e} p\left(\mathbf{y}_j \middle| \boldsymbol{\theta}_j, \sigma_j\right) \times p\left(\{\boldsymbol{\theta}_j\}_{j=1}^{N_e} \middle| \boldsymbol{\mu}, \boldsymbol{\Sigma}\right) \times p(\boldsymbol{\mu}, \boldsymbol{\Sigma}) \times \prod_{j=1}^{N_e} p\left(\sigma_j\right), \tag{7}$$

where $\boldsymbol{\mu}$, $\boldsymbol{\Sigma}$ are the hyperparameters of the hierarchical model representing the mean vector and covariance matrix of the individual "low-level" parameters and $\{\boldsymbol{\theta}_j, \sigma_j\}_{j=1}^{N_e}$ are the set of individual "low-level" parameters for each of the N_e repeats of the experimental recordings $\{\mathbf{y}_j\}_{j=1}^{N_e}$.

We sampled the full hierarchical Bayesian model using a simplified version of the Metropolis within Gibbs (24) method, which we have termed "pseudo-Metropolis within Gibbs" (see Fig. S9; Supporting Materials and Methods, Section S6, but note this simplification is only applicable for our particular setting, in which the number of data points in the time traces vastly outweighs the number of cells). We also describe the details of the choice of likelihoods and priors and sampling algorithms in Supporting Materials and Methods, Section S6, and we test the LogNormal distribution assumption in Supporting Materials and Methods, Section S8.

We used the inferred covariance matrix $\boldsymbol{\Sigma}$ to study the correlation ($\mathrm{corr}(\boldsymbol{\theta})$) between the model parameters, which are related by

$$\mathrm{corr}(\boldsymbol{\theta}) = \mathrm{diag}(\boldsymbol{\Sigma})^{-1/2}\, \boldsymbol{\Sigma}\, \mathrm{diag}(\boldsymbol{\Sigma})^{-1/2}, \tag{8}$$

where $\mathrm{diag}(.)^{-1/2}$ denotes the square root of the matrix of the diagonal entries. The posterior predictive distribution $p(\boldsymbol{\theta}|...)$ allows us to make predictions about how future experiments will behave, where (...) indicates all other variables appearing in Eq. 7. It can be computed using

$$p(\boldsymbol{\theta} \mid \cdots) = \int_{\boldsymbol{\Theta}} p(\boldsymbol{\theta} \mid \boldsymbol{\Theta}) p(\boldsymbol{\Theta} \mid \cdots)\, \mathrm{d}\boldsymbol{\Theta}, \tag{9}$$

where $\boldsymbol{\Theta} = \{\boldsymbol{\mu}, \boldsymbol{\Sigma}\}$, a concatenation of all the individual hyperparameters within $\boldsymbol{\mu}$ and $\boldsymbol{\Sigma}$. The integration was approximated by summing over the probability density functions, which are defined by the samples of $\boldsymbol{\Theta}$.

Synthetic data studies

Before implementing experiments, we confirmed the identifiability of model parameters using our protocols and parameter inference algorithms through a synthetic data study. We generated synthetic data (with added synthetic noise) with some known "true" parameters $\boldsymbol{\theta}^{\mathrm{true}}$. First, we used the synthetic data to design and optimize our protocols and to ensure that the protocols give access to sufficient information for parameter characterization. Second, we assessed our inference methods, described in the previous section, by asking how confident we are in our inferred parameters. In Supporting Materials and Methods, Section S6.3.1, we show that our newly designed protocol, the "staircase protocol" (see Fig. 1 *C*), is information-rich, in that we are able to fully recover the "true" parameter in a synthetic data study using our protocol.

We also tested our hierarchical Bayesian model to ensure that it is possible to infer the underlying distribution of the parameters. We generated our individual synthetic data from a predefined multivariate normal distribution, in which parameters are correlated. In Supporting Materials and Methods, Section S6.3.2, we applied our hierarchical Bayesian model analysis to the synthetic data, assuming we did not know the underlying covariance between parameters, and we were able to reconstruct the correlation matrix of our predefined distribution with very high accuracy. This provides us with confidence that our method is able to correctly infer the underlying correlation between parameters. We describe the rationale and procedure of the synthetic data study in detail in Supporting Materials and Methods, Section S6.

Experimental methods

Whole-cell patch-clamp voltage-clamp experiments were performed on Chinese hamster ovary (CHO) cells stably transfected with hERG1a (Kv11.1), with temperature control set to 25°C, using the Nanion SyncroPatch 384PE platform (Nanion Technologies). The temperature of the system's "cell hotel" was set to ~15°C. The machine is an automated high-throughput platform, in which each run (or chip) is able to measure up to 384 wells (with one cell per well) simultaneously. Single-hole chips with medium resistance (Nanion order number #221102) were used. Solutions used in all measurements are provided in Table S2.

A schematic of the experimental procedure is shown in Fig. 2, which shows the voltage-clamp protocols used in the experiments. A total of nine voltage-clamp protocols were used, including (*green*) our newly

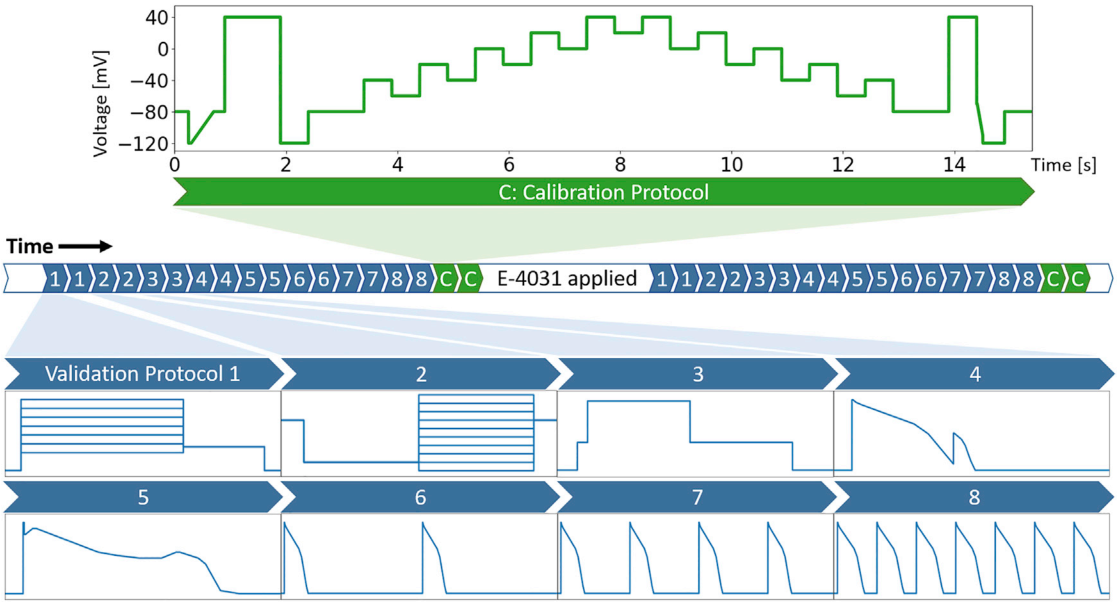

FIGURE 2 A schematic of the experimental procedure showing the sequence of voltage-clamp protocols used. A total of nine voltage-clamp protocols were used, and each of them was performed four times: twice before E-4031 addition and twice after to ensure stability and reliability of the recordings. Only the staircase protocol (*green*, 15 s) was used for fitting (or calibrating) the mathematical model. All of the other eight protocols (*blue*) were used for validation only. White sections indicate a nonmeasurement region, where cells were held at −80 mV to allow the cells to settle to steady state between protocols (>5 s) or were continuously stimulated by the hERG screening protocol to allow the drug to wash in (>5 min). For details of the protocols, please refer to Supporting Materials and Methods, Section S1. To see this figure in color, go online.

developed staircase protocol, (*blue*) an activation current-voltage (I-V) protocol, a steady-state inactivation I-V protocol, a hERG screening protocol, a delayed afterdepolarization (DAD)-like protocol, an early afterdepolarization (EAD)-like protocol, and action-potential-like protocols with beating frequency 0.5, 1, and 2 Hz, as shown in Fig. 2. Note that, because of the automated platform, the action-potential-like protocols have to be composed of a series of linear ramps and steps rather than curves. Details of the protocols are given in Supporting Materials and Methods, Section S1. Every protocol (the entire procedure in Fig. 2) was applied to every well. Because our quality control (see next section) is primarily based on the calibration recording, we decided to apply the calibration protocol at the end such that we can check the cell is stable for the entire experiment, including the validation protocols.

Only the staircase protocol (*green*) was used in fitting (or calibrating) the mathematical model. We show that we can fully characterize I_{Kr} for each cell using just this one protocol because our staircase protocol is information-rich. A comparison between the staircase protocol and a previously developed protocol (9) is shown in Fig. 1, *B* and *C*. However, because of hardware limitations, the previous protocol does not work in most high-throughput automated systems because they cannot perform clamps to arbitrary time-varying functions and are restricted to ramps and steps. Hence, a similar idea from Beattie et al. (9)—using an information-rich protocol—was adapted, and the rationale of our staircase protocol is discussed below. We designed the staircase protocol with only voltage steps and ramps such that it is applicable to any patch-clamp machine, including the high-throughput automated systems.

A demonstration that a mathematical model is able to reproduce the experimental training data is not sufficient to conclude that it is a good representation of ion channel kinetics—in particular, we may be uncertain how well the model performs under physiological conditions. The fitted models for each cell were therefore validated by comparison with experimental data from each of the other eight protocols (*blue* in Fig. 2). Our validation set consists of 1) two traditional I-V protocols together with a simple hERG activation step and 2) five physiologically inspired protocols that mimic cardiac action potentials. The first set allows us to compare with the traditional approach. More importantly, the second set allows us to have confidence in predictions of I_{Kr} responses, which is particularly useful when an ion channel model is embedded in a cardiac action potential model. This series of validations allows us to demonstrate that the models fitted using this new protocol yield trustworthy cell-specific predictions.

Protocol design

The underlying rationale of the staircase protocol shown in Fig. 2 is to force the protocol to explore the full dynamics of the system at different voltage values, over a physiologically relevant voltage range. By observing the changes in the current after each step, the voltage dependency of the channel at that particular voltage can be deduced. Each voltage step is held for 500 ms, which is chosen to be long enough to observe the characteristic decay of I_{Kr}. Therefore, by going through different step-ups and downs, the protocol explores the dynamics at different voltage values, and hence our statistical inference method is able to infer the underlying model parameters.

Two ramps are implemented before and after the main staircase. The ramp at the beginning, termed the "leak ramp," is used to estimate the leak current; see the next section for more details. The second one, happening after 14 s and termed the "reversal ramp," is designed to estimate experimentally the reversal potential E_K by having a ramp over 100 ms that quickly crosses the expected E_K, which we expect to be in the range of −70 to −100 mV. We therefore implemented a large step up to +40 mV before the ramp to open the channel so that we can record a high signal-to-noise ratio (SNR) I_{Kr} trace that goes from positive to negative before the channel closes. Examples of currents during the two ramps are shown in Fig. S2.

Postprocessing experimental data

We assumed that our observed current from hERG CHO cells under control conditions is

$$I_{\text{observed}}^{\text{control}} = I_{\text{Kr}} + I_{\text{endogenous}} + I_{\text{leak}}^{\text{a}}. \tag{10}$$

To ensure the currents we analyze are predominantly I_{Kr}, we performed a series of offline postprocessing corrections. First, leak corrections were applied to all measurements to eliminate the leak current $I_{\text{leak}}^{\text{a}}$. Second, E-4031 subtraction was applied to remove $I_{\text{endogenous}}$ (the sum of any native voltage-dependent ion currents that were present in CHO cells alongside the overexpressed hERG). These corrections are described in detail below, as well as our partially automated quality control criteria.

E-4031 subtraction

To eliminate any endogenous voltage-dependent background currents within the hERG CHO cells ($I_{\text{endogenous}}$ in Eq. 10), we measured the full set of nine voltage protocols twice (see Fig. 2); once with dimethyl vehicle conditions, in which $I_{\text{observed}}^{\text{control}}$ in Eq. 10 was measured, and once under the addition of 0.5 μM E-4031, a hERG channel selective blocker with IC50 value $\lesssim$10 nM, so that

$$I_{\text{observed}}^{\text{E-4031}} = I_{\text{endogenous}} + I_{\text{leak}}^{\text{b}}. \tag{11}$$

As shown in Fig. 2, a period of ~5 min was allowed for the E-4031 block to reach equilibrium, and multiple hERG screening protocols were applied to allow opening of the hERG channel (25). We denoted the new leak current as $I_{\text{leak}}^{\text{b}}$, and we assumed leak current changed over time; hence, in general, $I_{\text{leak}}^{\text{b}} \neq I_{\text{leak}}^{\text{a}}$. All currents shown or used in this study are the leak-corrected currents measured in control conditions minus the leak-corrected currents that remained after E-4031 addition, which we assume yields uncontaminated I_{Kr}.

Leak correction

We used the common assumption that leak current is linear in voltage to estimate its magnitude along the whole current trace

$$I_{\text{leak}}^{\text{est.}} = g_{\text{leak}}(V - E_{\text{leak}}), \tag{12}$$

where g_{leak} is the leak current conductance and E_{leak} is the leak current reversal potential. If we subtract an estimated leak off both $I_{\text{observed}}^{\text{control}}$ and $I_{\text{observed}}^{\text{E-4031}}$, then our final I_{Kr} can be given by

$$I_{\text{Kr}} \approx \underbrace{\overbrace{\left(I_{\text{observed}}^{\text{control}} - I_{\text{leak}}^{\text{est. a}}\right)}^{\text{Leak correction}} - \overbrace{\left(I_{\text{observed}}^{\text{E-4031}} - I_{\text{leak}}^{\text{est. b}}\right)}^{\text{Leak correction}}}_{\text{E-4031 subtraction}}, \tag{13}$$

where $I_{\text{leak}}^{\text{est. a}}, I_{\text{leak}}^{\text{est. b}}$ are leak currents estimated using Eq. 12. Depending on the protocol, we estimate the parameters g_{leak}, E_{leak} in one of two ways: either by using a step between two voltages or by using a linear ramp, as discussed below.

We assumed that at −80 mV, I_{Kr} is fully closed and will not be opened by going to a voltage below −80 mV. We therefore implemented the leak ramp: a linear ramp from −120 to −80 mV over 400 ms, as seen in the first second of the staircase protocol (*green*) in Fig. 2. All nonzero current measured during the leak ramp was assumed to be leak current in the form of Eq. 12, and a linear regression was used to fit its I-V relation and to obtain the leak model parameters. We show in detail the use of our leak ramp to infer the leak model parameters in Fig. S2, in which we can see that the recorded current during the ramp shows a reliably good linear relation. Therefore, this leak ramp can be used to check the linearity of the leak current, that is, the linearity in its I-V relation, which cannot be achieved using the standard voltage step method for leak estimation.

In a similar fashion, for all validation protocols, instead of a linear ramp, a traditional step method was used. A 20 ms leak step from −80 to −100 mV was used to leak-correct the experimental data. This method was implemented and performed automatically by the platform we used before every protocol to correct the recording that followed. However, we noticed that some of these leak corrections can "overcorrect" or "undercorrect" the current. For example, I_{Kr} should only be negative when the voltage is below its reversal potential, approximately −85.2 mV; if the leak-corrected current showed a negative current at voltages substantially larger than the reversal potential, then we concluded that the automated system had overestimated the leak current. Such overcorrection or undercorrection was most noticeable during the highest voltage step during the protocol, at which I_{leak} was at its maximum. For each validation protocol, we then specified a time window during which we believe I_{Kr} should be almost zero (please refer to our GitHub repository for detail). To rectify the over- or undercorrection, we re-estimated the leak correction by adding an extra linear leak current of the form $g_{\text{leak}}^{*}(V + 80\ \text{mV})$, where g_{leak}^{*} was chosen such that the average of the final leak-corrected current during the specified time window was zero. Because of the linearity, the final leak correction remains equivalent to Eq. 12 with different parameters.

Partially automated quality control

After the experiments, we applied a strict set of criteria as an automated selection process for quality control of our experimental data. The details of our criteria are summarized in Table 1. We applied a strict cutoff for seal resistance (R_{seal}), cell capacitance (C_m), and series resistance (R_{series}) through the whole set of measurements, set by our first quality control criterion (QC1). QC2 required a high signal-to-noise ratio (SNR) recording, such that our measurements contained enough useful information for model inference. We also compared the stability of the recordings in QC3, in which each protocol consisted of two measurements recorded in the same cell that must

TABLE 1 A Summary of the Fully Automated Quality Control Criteria for the Staircase Protocol, QC1–QC6

QC Name	Criterion Description
QC1.Rseal	Check R_{seal} within [0.1, 1000] GΩ.
QC1.Cm	Check C_m within [1, 100] pF.
QC1.Rseries	Check R_{series} within [1, 25] MΩ.
QC2.raw	Check raw trace recording SNR is over 25 (SNR defined as var(trace)/var(noise)).
QC2.subtracted	Check subtracted trace SNR > 25.
QC3.raw	Check 2 sweeps of raw trace recording are similar by comparing the RMSD of the two sweeps < mean(RMSD to zero of the two sweeps) × 0.2.
QC3.E4031	Check 2 sweeps of E-4031 trace recording are similar (same comparison as QC3.raw).
QC3.subtracted	Check 2 sweeps of subtracted trace recording are similar (same comparison as QC3.raw).
QC4…	Check R_{seal}, C_m, R_{series}, respectively, before and after E-4031 change (defined as std/mean) < 0.5.
QC5.staircase	Check the maximum current during the second half of the staircase changes by at least 75% of the raw trace after E-4031 addition.
QC5.1.staircase	Check RMSD to zero of staircase protocol changes by at least 50% of the raw trace after E-4031 addition.
QC6.subtracted	Check the first step up to + 40 mV, before the staircase, in the subtracted trace is bigger than −2× estimated noise level.
QC6.1.subtracted	Check the first + 40 mV during the staircase, with the same criterion as QC6.subtracted.
QC6.2.subtracted	Check the second + 40 mV during the staircase, with the same criterion as QC6.subtracted.

RMSD, root mean-square difference; SNR, signal-to-noise ratio; std, standard deviation; var, variance.

be similar and stable. QC4 required R_{seal}, C_m, and R_{series} to be stable before and after E-4031 addition. QC5 required that the addition of hERG blocker E-4031 must reduce a certain amount of the recorded current to ensure that our recordings consisted mainly of I_{Kr} even before adding the blocker. Finally, overcorrection of leak can occur during high voltage steps, as discussed in the previous section; QC6 ensured that no negative current occurred at voltages substantially larger than the reversal potential. Note that QC1 to QC4 are general criteria that are advocated to be used in all whole-cell patch-clamp voltage-clamp experiments, whereas QC5 and QC6 contain prior knowledge of I_{Kr} and are tailored to hERG measurements.

Using our automated high-throughput system, we recorded a total of 384 well recordings. Our automated quality control removed 173 wells, leaving 211 well recordings. We then manually checked all the recordings and subsequently removed a further 28 wells that did not look anything like the rest of the 183 cells; six typical examples are shown in Fig. S4. Therefore, our automated quality control has achieved >86% positive predictive value. The machine's "standard" quality control selects wells based mainly on the R_{seal}, C_m, and R_{series} values, and it was configured to use the same values as our automated QC1 in Table 1. The machine removed only 46 of the wells (which were all within our 173 discarded wells). Further comparisons and details of our automated quality control results are shown in Supporting Materials and Methods, Section S5. Our automated quality control is available at our GitHub repository.

Our mostly automated quality control was applied only to the staircase calibration protocol. In this study, we further require our validation data to contain high-quality validation recordings. We therefore manually selected 124 cells within our 183 cells that passed our quality control and hence have good recordings for both calibration and validation protocols; this ensures the quality of the experimental data used in this study. The overall success rate of recording our staircase protocol is 183 of 384 wells and for the full set of protocols is 124 of 384 wells, which can be performed within 1 h.

RESULTS

High-throughput experimental recordings

Fig. 3 shows the voltage-clamp recordings measured with the nine different protocols and the corresponding voltage protocols. All results shown are the first of the two repeats of our recordings. Our analysis was repeated for the second of the two repeats to ensure the reproducibility of our results in the same cells: the intrinsic (within-cell) variability is sufficiently small to appear negligible (see Fig. S12).

Fig. 3 *A* shows the staircase protocol (*black*) and the corresponding experimental recordings (*blue*). The middle panel shows the raw current recording of a single cell; the bottom panel shows the normalized current recordings from all 124 wells that passed quality control. Normalization is applied for visual comparison only because each hERG-transfected CHO cell is expected to have a different total conductance, hence giving a different magnitude of the current recorded. Currents are normalized by scaling them to minimize the absolute difference between each trace and a reference trace (*middle panel*). Because the reference trace is used only to normalize other traces for visualization, we simply picked a representative trace from our data that had reasonably low noise. Our recordings show a very similar result to the I_{Kr} simulation shown in Fig. 1, which used parameters calculated completely independently by Beattie et al. (9).

Fig. 3, *B–I* show the recordings of the other eight validation protocols from the same cells. The activation step in Fig. 3 *D* recorded a typical I_{Kr} response, in which the step-down of voltage to −40 mV largely opens the channels. Fig. 3, *G–I* also show typical I_{Kr} responses to the action-potential clamp at different pacing frequencies, at which I_{Kr} is active during repolarization of the action potential. Also note the sharp opening of I_{Kr} at the upstroke that changes with pacing frequencies and increases dramatically but very consistently across all the recorded cells.

Individual cell fitting and validation

Fig. 4 shows the same voltage-clamp recordings (*blue*) in Fig. 3, measured under the nine different protocols (*black*), together with model fitting and validation results. All recordings shown were performed on a single cell. The mathematical model, shown as red lines, is fitted only to the data recorded under the staircase protocol that is shown in Fig. 4 *A*. The result of the fitting for a single cell is shown in the middle panel of Fig. 4 *A*, demonstrating an excellent fit between experimental measurement and simulated current. The inferred parameters are shown and studied in detail in the next three sections.

In Fig. 4, *B–I*, we show the results of the validation predictions under eight other protocols. We validated our trained model by testing its ability to predict independent experimental outcomes under different protocols, which were measured in the same cell. All validation predictions were performed by using the inferred parameters in the fit to the staircase protocol (Fig. 4 *A*) to simulate the other eight protocols (Fig. 4, *B–I*). The predictions of all the protocols match very well to the experimental data, with the simulated currents giving a close match to the experimental recordings.

The physiologically inspired voltage-clamp protocols (Fig. 4, *E–I*) mimic the membrane voltage of the cardiac action potential at normal conditions at different beating rate and EAD/DAD-like conditions. The ability to predict the current response under these physiologically inspired voltage-clamp protocols is particularly important for use in physiological or pharmacological studies. This shows the reliability of the hERG ion channel model predictions at different physiological conditions, for example, when it is embedded in a whole-cell cardiac model for further predictions.

In Fig. 5, we present our model fitting and validation results for all 124 cells, compared against the experimental recordings measured under the nine different protocols. We applied the same fitting and validation procedure as used for the single cell discussed above to all 124 cell measurements. To visualize the variability in only hERG kinetics (and not maximum conductance), we plotted all currents normalized as described in the previous section.

We quantified the fits and predictions using relative root mean-square error (RRMSE), defined as the root mean-square

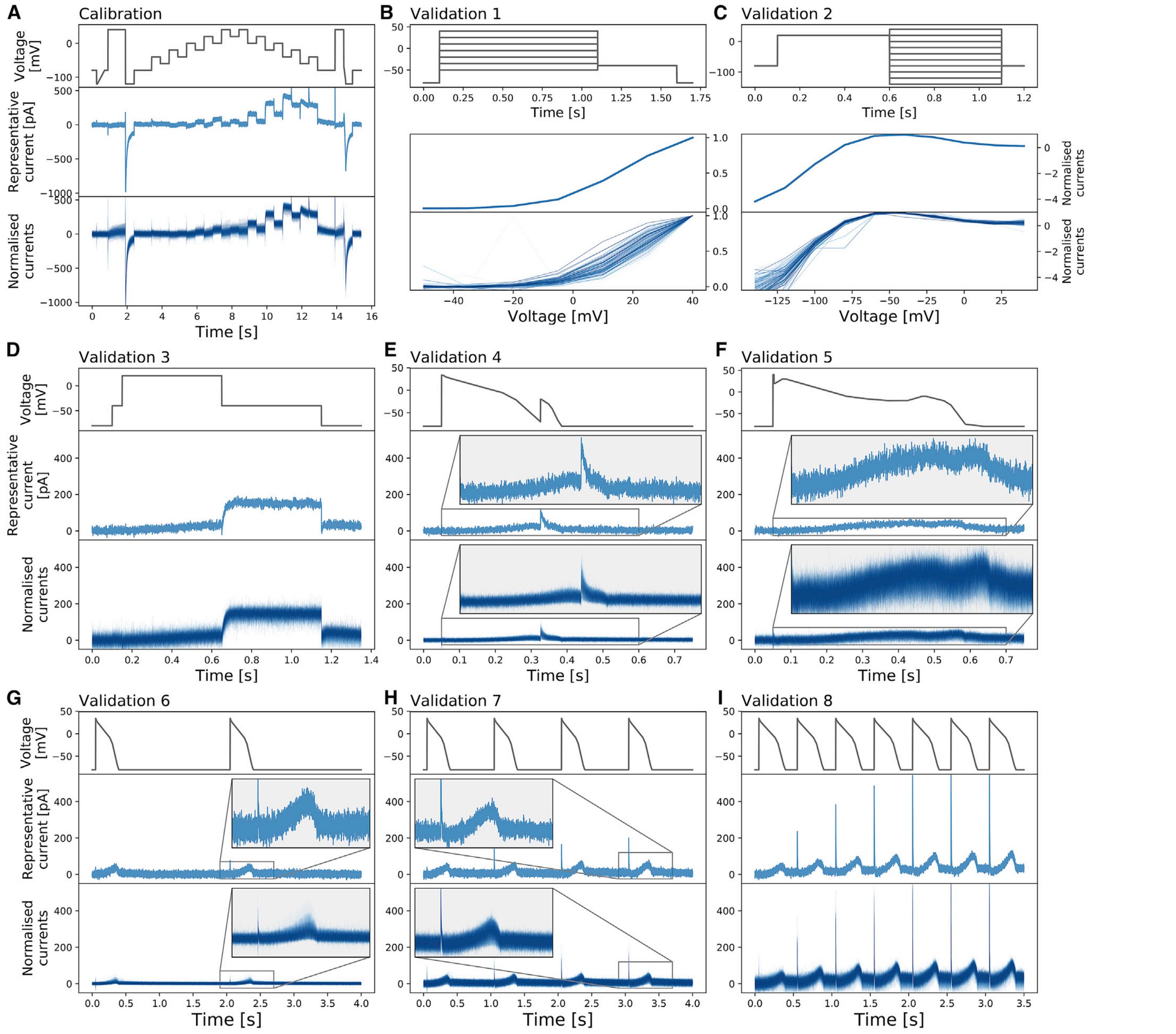

FIGURE 3 Whole-cell patch-clamp voltage-clamp recordings under nine different protocols, which were all measured in each cell. (*A*) shows the staircase protocol (*top panel*) in black and the corresponding recording on a single cell (*middle panel*) and normalized recordings from all 124/384 wells that passed quality control (*bottom panel*) in blue. Conductance normalization was done by multiplying each current by a scaling factor to minimize the absolute difference between each trace and a reference trace (*middle panel*). (*B–I*) The eight different protocols used as validation of the model calibration, which are the activation current-voltage (I-V) protocol, the steady-state inactivation I-V protocol, the hERG screening protocol, the DAD-like protocol, the EAD-like protocol, and the cardiac action-potential-like protocol at 0.5, 1, and 2 Hz, respectively, are shown. All experimental recordings, both the single-cell (*middle*) and 124 cells (*bottom*), are shown in blue, which were measured under the protocol (*black*) shown in the panels immediately above. In (*B*) and (*C*), validation 1 and 2 show the I-V relations extracted from the currents. To see this figure in color, go online.

error between the model simulation and the experimental data, divided by the root mean-square distance of the data to a zero-current trace:

$$\mathrm{RRMSE} = \sqrt{\sum \left(I_{\mathrm{Kr}}^{\mathrm{model}} - I_{\mathrm{Kr}}^{\mathrm{data}}\right)^2 \Big/ \sum \left(I_{\mathrm{Kr}}^{\mathrm{data}}\right)^2}. \quad (14)$$

Using this RRMSE quantification, the difference in the absolute size of the current across cells due to varying conductance is eliminated, and RRMSE scores are comparable between cells. Fig. 6 shows the RRMSE histograms for all cells and for six of the protocols. Markers indicate the best (*), median (‡), and 90th percentile (#) RRMSE values, and corresponding raw traces are shown in the three

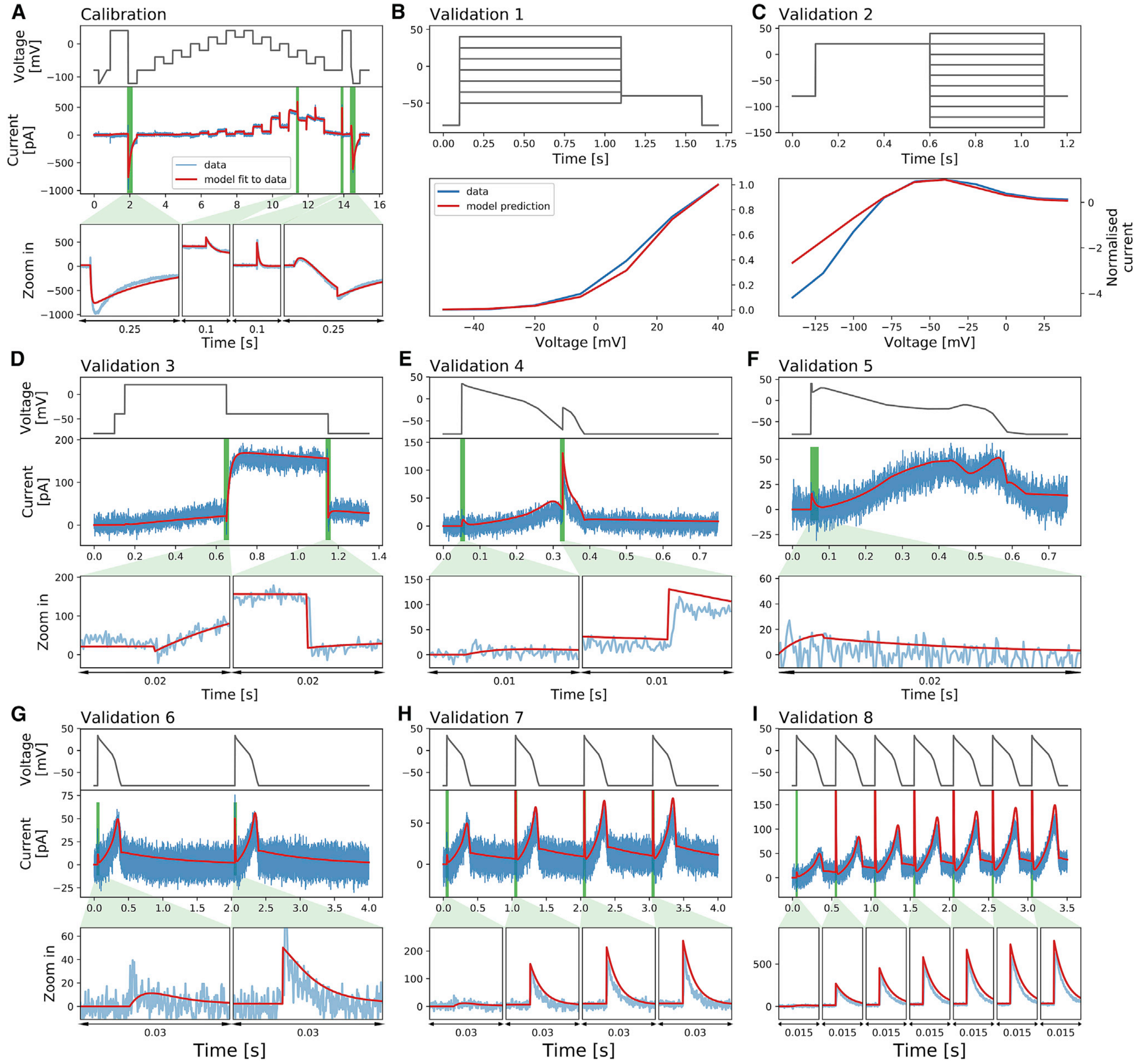

FIGURE 4 Whole-cell patch-clamp voltage-clamp recordings under nine different protocols that were measured on a single cell and the model fitting and validation results. (*A*) shows the staircase protocol (*black*) and the corresponding recording (*blue*). The mathematical model is calibrated using this recorded data, and shown as a red line. (*B–I*) The eight different protocols used as validation of the calibrated model, which are the activation I-V protocol, the steady-state inactivation I-V protocol, the hERG screening protocol, the DAD-like protocol, the EAD-like protocol, and the cardiac action-potential-like protocol at 0.5, 1, and 2 Hz, respectively, are shown. All experimental recordings are shown in blue, which were measured under the protocol (*black*) shown in the panels immediately above, and the validation predictions of the model are shown in red. Zoomed-in image of the green shaded regions are shown underneath each panel to reveal the details of the spikes, in which our model also shows excellent predictions of the faster timescale behavior. In (*B*) and (*C*), validation 1 and 2 show I-V relations extracted from these protocols. To see this figure in color, go online.

panels above. The solid diamond marker (◆) indicates the reference cell shown in Figs. 3 and 4. The same analysis applied to the remaining three protocols is shown in Fig. S15. There are some small discrepancies in the predictions, for example, in Fig. 6 *B* in the 90th percentile predictions. But overall, these results demonstrate that all our 124 models make very good predictions for the recorded current kinetics.

Next, we first qualitatively inspect the variability in the hERG kinetics measurements. Because we measured the I_{Kr} using exactly the same experimental setup for each cell, we can clearly see the variability between measurements in all of the recordings, as illustrated in Fig. 5. Different protocols demonstrate different levels of variation. It is clear that among the six protocols, the staircase protocol and the two I-V protocols show the strongest variation between measurements.

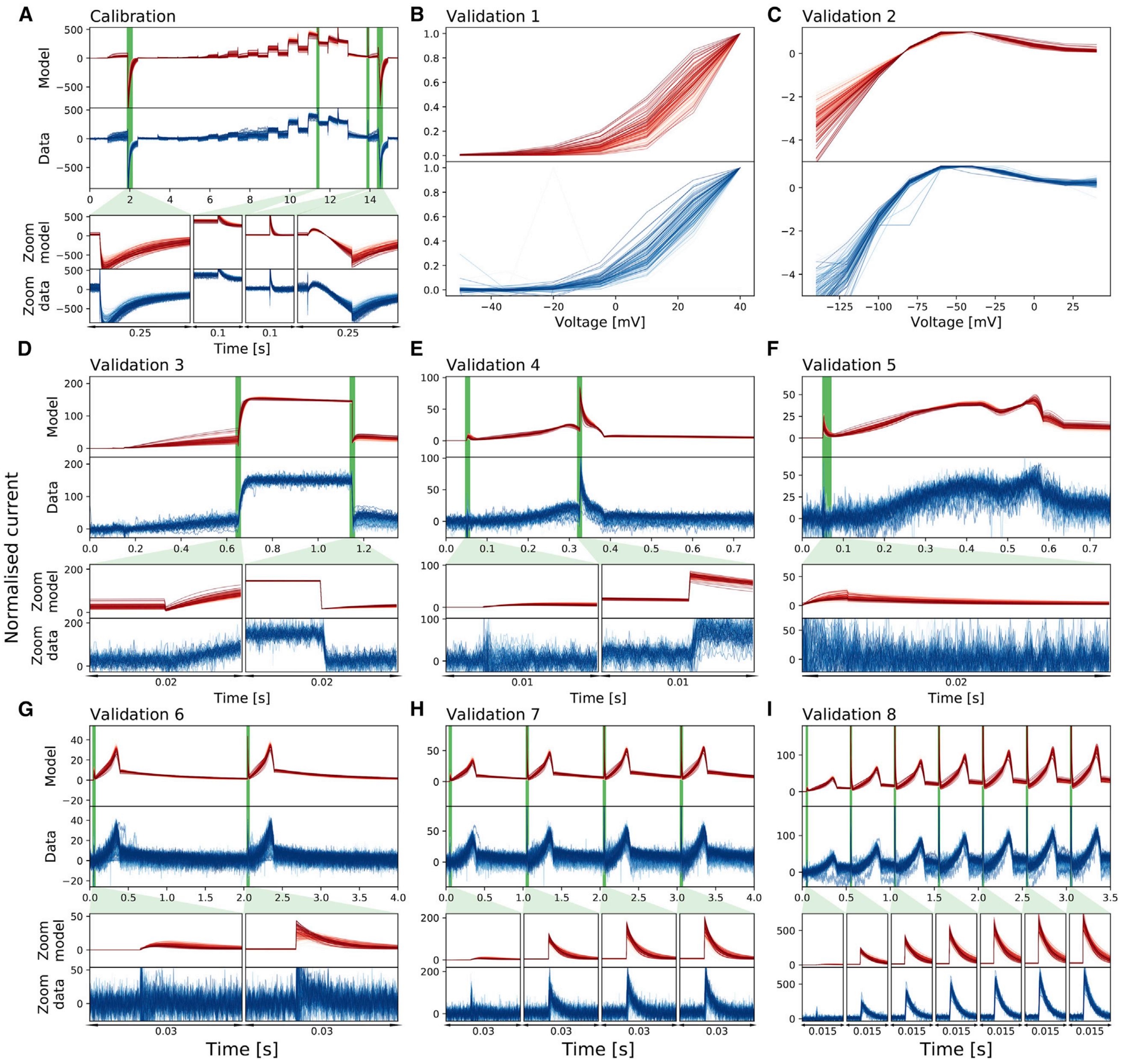

FIGURE 5 Normalized whole-cell patch-clamp voltage-clamp recordings for 124 cells under nine different protocols and the model fitting and validation results. All currents are normalized by scaling them to minimize the absolute difference between each trace and a reference trace. From (*A*) to (*I*): the staircase protocol which is used as the calibration protocol, the activation I-V protocol, the steady-state inactivation I-V protocol, the hERG screening protocol, the DAD-like protocol, the EAD-like protocol, and the cardiac action-potential-like protocol at 0.5, 1, and 2 Hz, respectively, are shown. All the model calibration results and validation predictions are shown in the top panels (*red*) and are compared against the experimental recordings shown in the bottom panels (*blue*). Magnifications of the green shaded regions are shown underneath each panel to reveal the details of the spikes, in which our models show extraordinarily good predictions to the details. The normalized current for all protocols is shown except for the activation I-V protocol and the steady-state inactivation I-V protocol, in which the summary statistic I-V relationships are shown. To see this figure in color, go online.

To investigate this further, we have used our mathematical model to study the variability in the parameter values that could drive the observed variability in the outputs. Fig. 7 shows the inferred parameter values which are used in the model predictions in Fig. 5. Because we assume all cells share the same mechanistic model underlying the hERG currents our inferred cell-specific model parameters capture the cell-to-cell variability, or rather, experiment-to-experiment variability. In Fig. 7, our inferred parameters are plotted against manual patch parameters (shown as *orange dots/red squares*), measured at a slightly lower (room) temperature, from Beattie et al. (9); our identified parameters are broadly in alignment with manual patch results. This agreement gives us

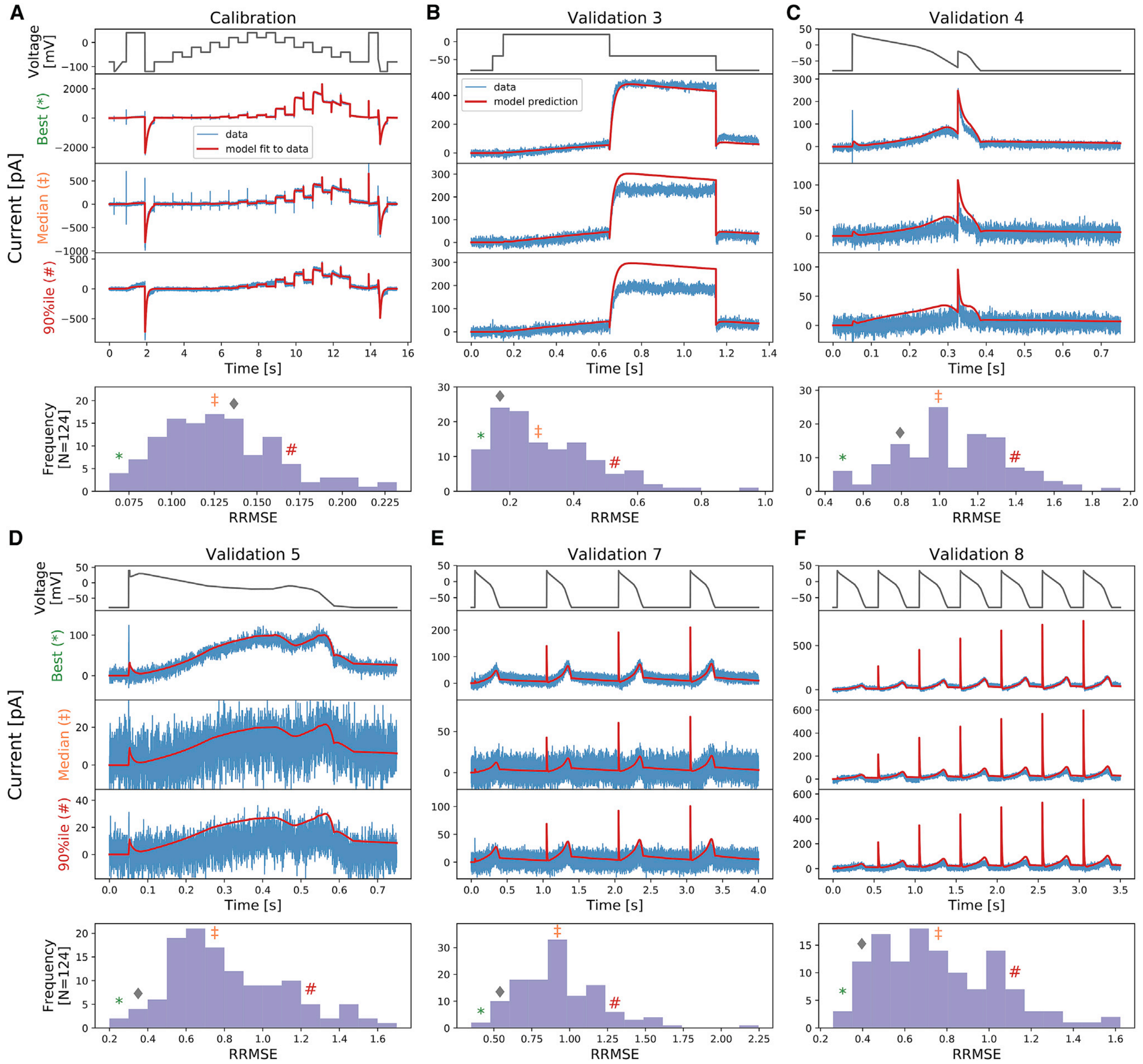

FIGURE 6 The relative root mean-square error (RRMSE, given by Eq. 14) histograms for six protocols (*A–F*). Each histogram represents the same 124 cells with a different protocol and RRMSE each time. Markers indicate the best (*), median (‡), and 90th percentile (#) RRMSE values, and the solid diamond marker (◆) indicates the error for the reference traces shown in Figs. 3 and 4. For each protocol, the raw traces with the best, median, and 90th percentile RRMSE values for both the model (*red*) and data (*blue*) are shown, with the voltage clamp above. Note that the currents are shown on different scales to reveal the details of the traces. The same analysis applied to the remaining three protocols is shown in Fig. S15. To see this figure in color, go online.

further confidence that our high-throughput method is reproducible and biophysically meaningful. We can also see that there is more variability in some parameters than others, also seen in the previous study (9). In particular, p_1, p_3, and p_6 show stronger variability that varies over an order of magnitude, whereas the others vary only within an order of magnitude. Conductance g_{Kr} also varies significantly, but this might be expected given different sizes and expression levels for individual cells.

A hierarchical Bayesian modeling approach to characterize well-to-well variability

We applied the hierarchical Bayesian model to analyze the variability within the experimental recordings and correlations between inferred well-to-well parameter sets. The result of applying our hierarchical model is shown in Fig. 8. The measurement uncertainty for the parameters of each individual well is shown with a marginal posterior distribution, the colored histograms. Most of the parameters

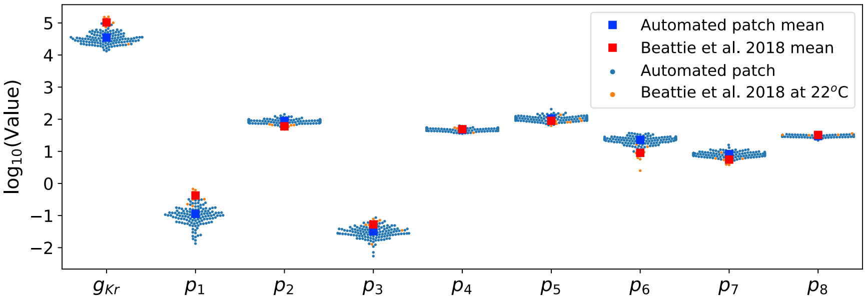

FIGURE 7 Cell-specific model parameters at around 25°C. The inferred parameter values shown here are obtained from the staircase protocol calibration and are also the parameters used in the model predictions in Fig. 5. It also shows the manual patch obtained parameters (*orange*), measured at around 22°C, from Beattie et al. (9). The inferred kinetic parameter values from the automated high-throughput system are broadly consistent with the manual patch measurements. To see this figure in color, go online.

give a narrow credible interval, which reinforces our certainty in the information content of the calibration protocol. Many of the marginal posterior distributions of the individual wells overlap, that is, we cannot distinguish between the two sets of parameters given our uncertainty in them. However, some of the individual marginal posterior distributions are distinct from each other, demonstrating considerable variability between wells.

The power of the hierarchical Bayesian model can then be used to summarize and capture the experiment-to-experiment variability. The hyperparameters of the model describe both the mean $\boldsymbol{\mu}$ and (co)variance $\boldsymbol{\Sigma}$ of parameter sets across wells, with experimental uncertainty taken into account. We estimated this posterior predictive distribution (Eq. 9) from the samples of hyperparameters, and its marginal distributions are shown for each parameter as the red curves in Fig. 8. This distribution can be used to predict the likelihood and variability of parameter sets from further wells in future experiments. The mean values of the samples of $\boldsymbol{\mu}$ (which is equivalent to the mean of the posterior predictive distribution) and its 95% credible intervals are provided in Table S3. To compare with previous literature values, we plotted the activation and inactivation steady-state curves a_∞ and r_∞ from these parameter sets alongside results from Sanguinetti et al. (1), resulting in a good correspondence shown in Fig. S18.

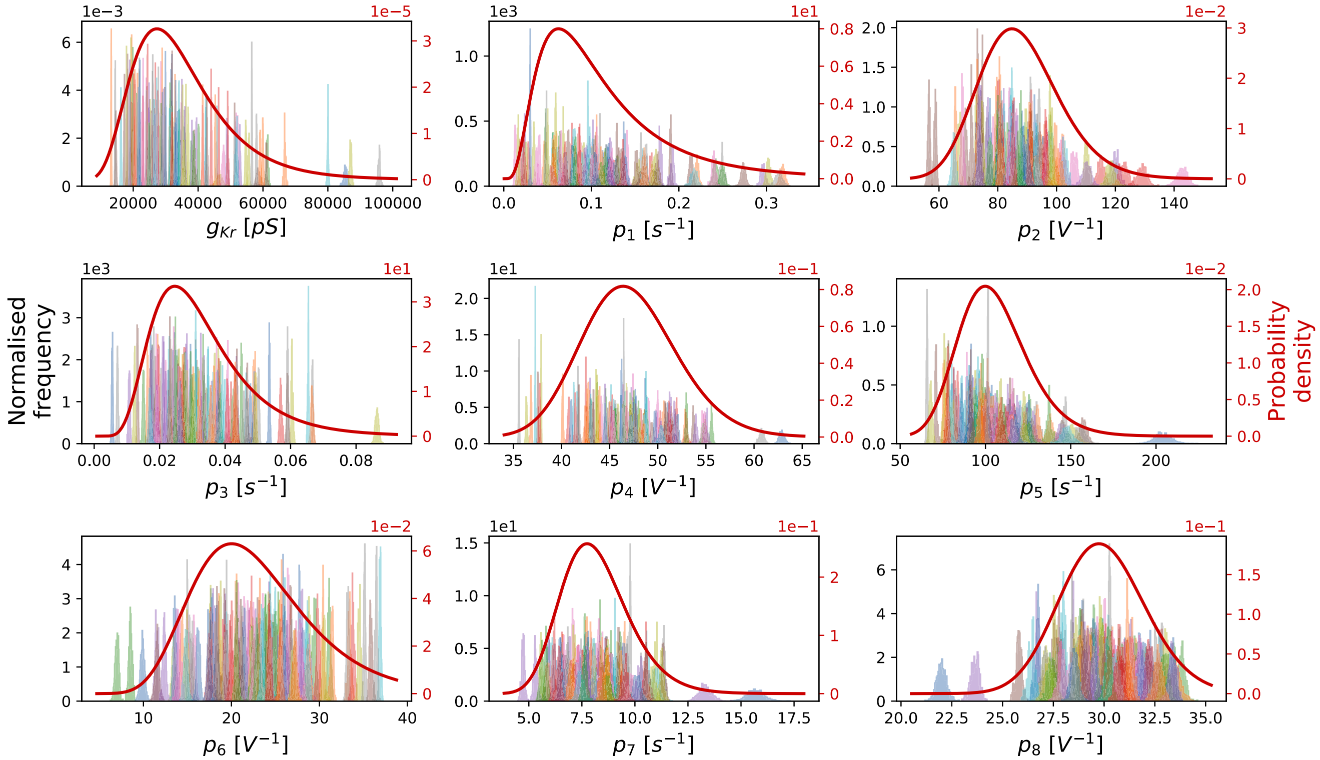

FIGURE 8 The marginal distributions from the hierarchical Bayesian model for all model parameters. Left *y* axis: individual histograms show the normalized marginals (probability densities) for each parameter in each well, with different colors representing the 124 individual wells. Each of them shows a narrow distribution, which implies good confidence in our inferred parameters for the individual well. Right *y* axis: red curve shows the marginal posterior predictive distributions across cells $p(\boldsymbol{\theta}|...)$, which are assumed to follow a multivariate log-normal distribution for each parameter. They show the inferred underlying distribution across cells for each of the parameters. To see this figure in color, go online.

Finally, we utilized the hierarchical Bayesian model to investigate the correlation between model parameters across different wells. In the sampled hyperparameters, the covariance matrix $\boldsymbol{\Sigma}$ reveals any correlation between our model parameters. The typical assumption concerning the variability of parameters is that parameters are independent, i.e., in the covariance matrix, all entries except the diagonal are zero. In the upper triangle (*orange*) of Fig. 9, we compare our inferred correlation between parameters (calculated using Eq. 8) with this common assumption (*black vertical dashed lines*). It is obvious that there are many entries in which zero is outside our credible interval, which is equivalent to showing that the independence assumption is not supported by our findings.

To visualize the correlation between parameters better, the 95% credible regions for each pair of parameters are shown in the lower triangle (*blue*) of Fig. 9, plotted against the scatter plot of the 124 cells' individual posterior mean parameters (shown on a log-scale). Each blue ellipse is reconstructed from a sample of hyperparameters, where the contour of the 95% credible region of the two-variate marginal distribution defined by the hyperparameter sample is shown, capturing most of our individual posterior mean parameters appropriately. In this plot, a perfect circle implies there is no correlation between the pair of parameters. However, we can clearly see that most of our pairwise parameters show an elliptical shape, which means some degree of correlation between the pairwise parameters exists. This strongly suggests that correlations between parameters are embedded in the experiment-to-experiment variability. To ensure our observed correlations are biophysically relevant rather than a sign of identifiability problems, in Supporting Materials and Methods, Section S10, two figures show how cell-specific parameters make accurate cell-specific predictions. We further discuss explanations for such observed correlations in the Discussion.

DISCUSSION

In this study, we have developed a short, high-information-content staircase voltage-clamp protocol for I_{Kr} that is applicable in automated high-throughput patch-clamp systems and used a mathematical model to characterize channel kinetics by fitting its parameters to recordings made under this new protocol. This study will advance future ion channel model development and model selection and forms a basis for improved screening of ion channel kinetics under different conditions, mutations, or pharmaceutical compounds.

Here, we no longer use I-V or τ-V relations to characterize hERG kinetics, but rather, we use a mechanistic model and its parameterization to capture our knowledge of channel kinetics. An optimized voltage protocol, which is short and has a high information content, was used to parameterize the hERG kinetics model. The benefits of this approach are threefold. First, current "rundown" during the protocol has less of an effect over shorter experiments (tens of seconds) as compared to traditional I-V and τ-V protocols (tens of minutes); hence, it is much easier to obtain a measurement that remains stable. Second, given its short duration, it is easy to repeat the measurement to examine within-cell reproducibility and/or variability. Third, our staircase protocol can be used to rapidly create cell-specific models of kinetics (which is much harder to do using the more time-consuming traditional I-V and τ-V protocols).

We have shown that our 15 s staircase protocol can be performed in an automated high-throughput system. We have found that each of the resulting 124 models is consistent with previous manual patch-clamp results (limited to nine cells) (9), implying that these methods are reproducible. We can now easily produce large data sets for further analysis, which is usually difficult, if not impossible, to achieve with manual patch clamp. The predictions of the cell-specific models are not perfect, as we examined in Fig. 6, and there may be room for improvement in terms of the model structure and further optimization of the calibration protocol. But we are able to calibrate our model to the extent that it can replicate both experimental training data and predict validation data very well (Fig. 4). Our models can predict the current response to the physiologically relevant action potential protocols, demonstrating that our I_{Kr} models could be useful in predicting cardiac electrical activity in both healthy and arrhythmic situations (9). This provides assurance that our cell-specific models, which are constructed in a high-throughput manner, have great potential for future uses.

For example, our method can potentially be adapted and used to investigate not only how much the hERG channel is blocked by a drug but also how that drug influences channel kinetics. This might be useful for the Comprehensive in vitro Proarrhythmia Assay initiative because both automated high-throughput systems and in silico modeling constitute the core of the initiative (26,27). Our approach may give us a better understanding of the pharmacological properties of drugs in the screening process and hence a better pharmaceutical safety assessment. We can also incorporate the cell-to-cell or experiment-to-experiment variability in the in silico modeling as part of the uncertainty quantification for safety-critical predictions (15). Furthermore, such rapid characterization using high-throughput systems can benefit precision and personalized medicine. For example, when using human-induced pluripotent stem cell-derived cardiomyocytes, as described in Lei et al. (28), characterization of ion current kinetics may need to be taken into account to tailor accurate cell-line-specific models.

With our 124 cell-specific hERG models, we are able to study experiment-to-experiment variability in the hERG channel. Such experiment-to-experiment variability is captured using our hierarchical Bayesian model, in which the posterior

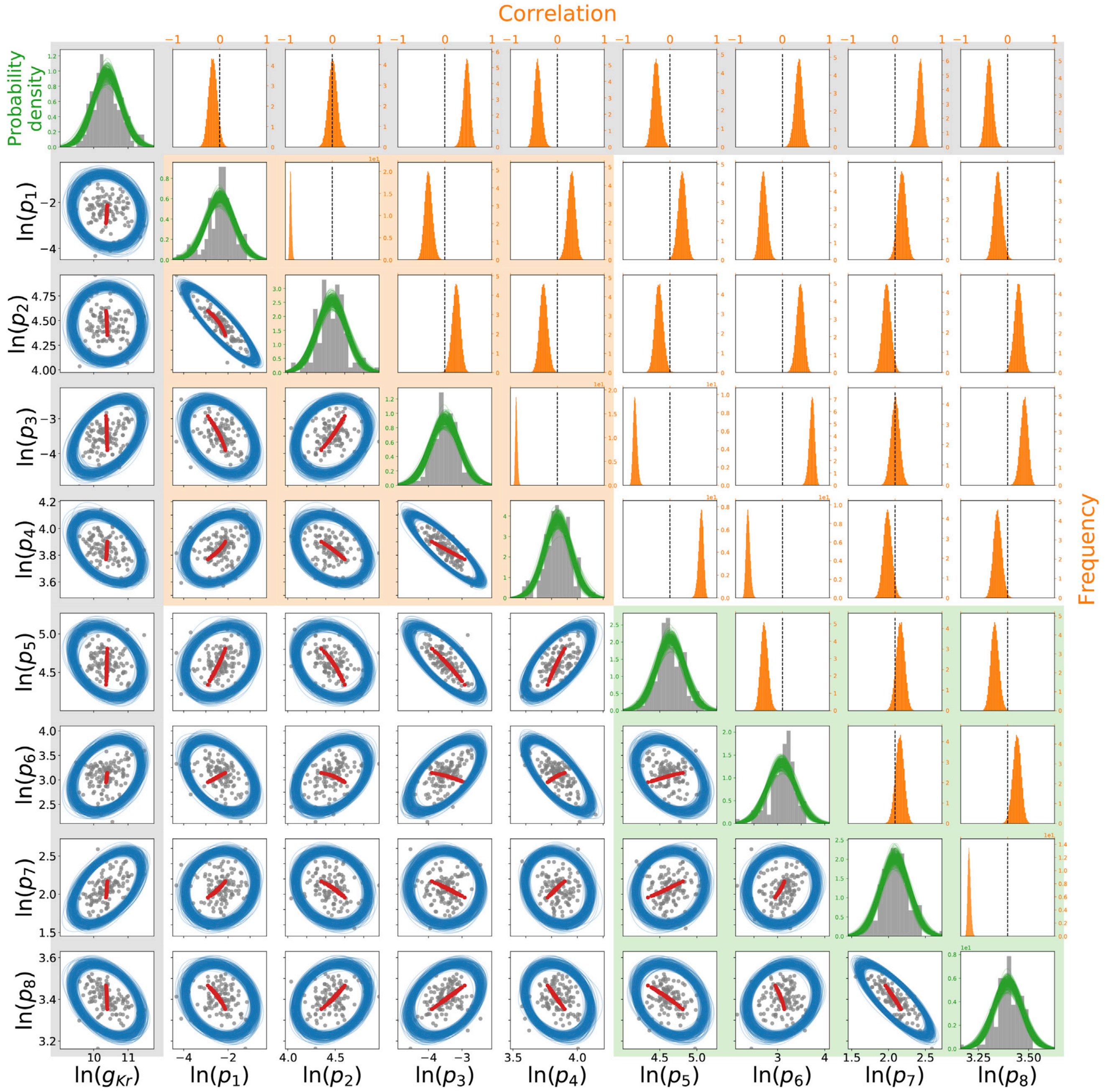

FIGURE 9 The inferred correlation in model parameters across experimental wells. All parameters shown here are natural log-transformed. The posterior mean parameters ($\boldsymbol{\theta}$) of each of the 124 individual wells are shown in gray (*dots* and *histograms*). Note that the posterior distributions for each well are so tight that only the mean values are shown for clarity (see Fig. 8); full example posteriors for a pair of cells are shown in Fig. S16. Lower triangle (*blue*): the 95% credible region boundary for the distribution of parameters across wells is shown. Each credible region ellipse is reconstructed from one sample of the $\boldsymbol{\mu}$, $\boldsymbol{\Sigma}$ across-cell distribution parameters from the MCMC chain of size 10^5; for clarity, only 200 samples are shown here. Simulated voltage error offset (described in the Discussion) is shown as red dots. Diagonal (*green*): the sampled posterior probably density functions before integration to give $p(\boldsymbol{\theta}|...)$ are given, shown in detail in Fig. 8. Upper triangle (*orange*): the marginal histograms for each entry of the correlation matrix defined by Eq. 8 are given. The common assumption of independence (correlation of zero) is shown as black vertical lines for comparison. The shadings in the background indicate how these parameters relate to the model structure: the orange box contains the gates *a* in model, green box contains gate *r*, and gray relates to the conductance. To see this figure in color, go online.

predictive distribution is constructed and describes the underlying variability of the parameters (Fig. 8). Instead of using a series of I-V and time constant-voltage curves, here, we evaluate the variability of the observed hERG channel kinetics using mathematical model parameters. The variability in the parameter values predicts the observed differences in the channel kinetics; see also Supporting Materials and Methods, Section S10. In addition, we can use our posterior predictive

distribution to predict what might happen in future experiments based on the observed experiments.

Sources of variability

We have successfully quantified the variability between wells via our inferred model parameters. However, the underlying cause of this variability is an open question. There are possibilities at two extremes. One is that the variability is truly cell-to-cell and ion channel kinetics do vary because of different intracellular conditions, which one may speculate is due to differing gene expression, subunits, phosphorylation states, or suchlike. The other possibility is that ion channel kinetics are precisely identical in each cell but there are some experimental artifacts, varying between wells, that are causing the observed variability in parameters from each well. Below, we discuss hints in our results as to which of these extremes is the leading cause of variability.

As mentioned in the rationale of the staircase protocol, the 100 ms reversal ramp at 14.41 s was introduced to estimate experimentally the hERG reversal potential E_K^j in each of the j wells; for details, see Fig. S2. Fig. 10 shows an example E_K^j derivation using the reversal ramp and a histogram of E_K^j values estimated from the 124 wells.

Our obtained histogram of E_K^j values is distributed close to our theoretical E_K from the Nernst equation (Eq. 2), with an SD of 1.36 mV. Because all of our measurements were performed on one 384-well plate, they shared the same extra- and intracellular solutions and were recorded at (almost) the same temperature. We would therefore expect the real variability in reversal potential to be much smaller than this observed variability.

A hypothesis then, is that reversal potential E_K really occurs at the Nernst calculated value, and observed deviations from this inferred from the reversal ramp provide an estimate for a "voltage error" in the applied voltage clamp: $\Delta V^j = E_K^j - E_K$, perhaps due to an imperfect compensations of other external effects, such as junction potentials and electrode offsets. We can investigate this hypothesis via the model by applying a staircase protocol with voltage error offsets of ΔV^j estimated from each of the 124 cells, generating synthetic data from these voltage clamps, and then refitting parameters.

Fig. 9 (*lower triangle*) shows the results of our voltage error offset simulations in red dots. If there was an error in the applied voltage clamp in each well, then we would expect to see parameters appearing to covary along the red lines (made up of individual *dots/fits*) in Fig. 9. The observed primary parameter covariance directions and magnitudes from this procedure (*red lines*) align suspiciously well with much of the observed variability in the experiments (*blue ellipses* inferred from *gray dots*). In Supporting Materials and Methods, Section S13, we extend Fig. 9 (*lower triangle*) to show the value of ΔV^j for each individual well j, which indeed finds a correlation along the proposed directions, further supporting the hypothesis.

This is strong circumstantial evidence—a smoking gun—suggesting that the majority of the observed variability in parameters may be due to well-well variability in patch-clamp artifacts rather than cell-cell variability in ion channel kinetics. We explored the possibility of the quality control parameters having direct bearing on the estimated voltage error; however, no obvious correlation between these values is apparent, as shown in Fig. S19. Building a more complete mathematical model of such patch-clamp artifacts is part of our future plans. We should also note that despite patch-clamp artifacts being an apparent cause of parameter variability, they are not necessarily larger artifacts in this automated system than might be expected in manual patch clamp.

Finally, if we were to believe that the observed variability here arises from experimental artifacts, then only the uncertainty in the top-level mean parameter vector $\boldsymbol{\mu}$ in the hierarchical Bayesian model is representative of our uncertainty in the underlying physiology. That is, the variability of top-level mean parameter vector $\boldsymbol{\mu}$ should be included in future physiological studies, for example, in the second part of this study (this issue of *Biophysical Journal*, Lei et al. (29)) or when embedding an ion channel model within an action potential model, whereas the full posterior predictive distribution should be used only when predicting the results of future patch-clamp experiments.

CONCLUSIONS

In this study, we have demonstrated the feasibility and practicality of using a 15 second staircase protocol to

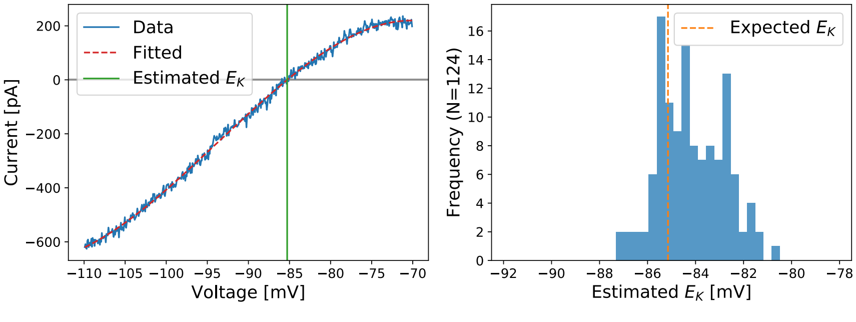

FIGURE 10 Left: an example of the current-voltage relationship plotted for the last ramp in the staircase protocol and how it is used to estimate the E_K reversal potential value for I_{Kr} in one well. Right: a histogram of E_K values estimated using the reversal ramp technique is shown. The E_K values here were estimated from the same 124 wells used in the main results. The dashed orange vertical line shows the expected E_K calculated directly from temperature and concentrations using the Nernst equation (Eq. 2). To see this figure in color, go online.

study and characterize hERG channel kinetics on an automated high-throughput system. We calibrated the hERG model to our staircase protocol for 124 hERG cells. Our 124 cell-specific variants of the hERG model are able to predict eight other protocols with a high accuracy, including physiologically inspired action-potential-like voltage clamps. Using a hierarchical Bayesian modeling approach, we provide a quantitative description of the variability and uncertainty within our 124 cell-specific models.

With our rapid characterization techniques and the hierarchical Bayesian modeling approach, we have opened a, to our knowledge, new gateway to study parameter correlations between cells and investigate experimental variability. We have found that some model parameters are strongly cross-correlated, but not all. This result may hint at the origin of the variability and requires further investigation. In future, we aim to design protocols to allow high-throughput systems to be used to investigate not only how much the hERG channel is blocked by a drug but also the kinetics of drug binding and whether the drug influences underlying channel kinetics.

All codes and data are freely available at https://github.com/CardiacModelling/hERGRapidCharacterisation, a permanently archived version is available on Figshare at https://doi.org/10.1016/j.bpj.2019.07.030.

SUPPORTING MATERIAL

Supporting Material can be found online at https://doi.org/10.1016/j.bpj.2019.07.029.

AUTHOR CONTRIBUTIONS

C.L.L., M.C., D.J.G., L.P., G.R.M., and K.W. designed the research. C.L.L., L.P., and K.W. carried out the experiments. C.L.L., M.C., D.J.G., and G.R.M. designed the computational analysis. C.L.L. wrote simulation codes, performed the analysis, and generated the results figures. All authors wrote and approved the final version of the manuscript.

ACKNOWLEDGMENTS

We thank Nanion for assistance with automating the export of current trace data.

This work was supported by the Wellcome Trust (grant numbers 101222/Z/13/Z and 212203/Z/18/Z), the Engineering and Physical Sciences Research Council and the Medical Research Council (grant number EP/L016044/1), and the Biotechnology and Biological Sciences Research Council (grant number BB/P010008/1). C.L.L. acknowledges support from the Clarendon Scholarship Fund; and the Engineering and Physical Sciences Research Council, Medical Research Council, and F. Hoffman-LaRoche Ltd for studentship support via the Oxford Systems Approaches to Biomedical Science Centre for Doctoral Training. M.C. and D.J.G. acknowledge support from a Biotechnology and Biological Sciences Research Council project grant. G.R.M. acknowledges support from the Wellcome Trust and Royal Society via a Sir Henry Dale Fellowship and a Wellcome Trust Senior Research Fellowship. L.P. and K.W. are employees of F. Hoffman-LaRoche Ltd., and K.W. is a shareholder.

REFERENCES

1. Sanguinetti, M. C., C. Jiang, ..., M. T. Keating. 1995. A mechanistic link between an inherited and an acquired cardiac arrhythmia: HERG encodes the IKr potassium channel. *Cell.* 81:299–307.
2. Jurkiewicz, N. K., and M. C. Sanguinetti. 1993. Rate-dependent prolongation of cardiac action potentials by a methanesulfonanilide class III antiarrhythmic agent. Specific block of rapidly activating delayed rectifier K+ current by dofetilide. *Circ. Res.* 72:75–83.
3. Malik, M., and A. J. Camm. 2001. Evaluation of drug-induced QT interval prolongation: implications for drug approval and labelling. *Drug Saf.* 24:323–351.
4. Friedrichs, G. S., L. Patmore, and A. Bass. 2005. Non-clinical evaluation of ventricular repolarization (ICH S7B): results of an interim survey of international pharmaceutical companies. *J. Pharmacol. Toxicol. Methods.* 52:6–11.
5. Mirams, G. R., Y. Cui, ..., D. Noble. 2011. Simulation of multiple ion channel block provides improved early prediction of compounds' clinical torsadogenic risk. *Cardiovasc. Res.* 91:53–61.
6. Li, Z., B. J. Ridder, ..., D. G. Strauss. 2019. Assessment of an in silico mechanistic model for proarrhythmia risk prediction under the CiPA initiative. *Clin. Pharmacol. Ther.* 105:466–475.
7. Vanoye, C. G., R. R. Desai, ..., A. L. George, Jr. 2018. High-throughput functional evaluation of KCNQ1 decrypts variants of unknown significance. *Circ Genom Precis Med.* 11:e002345.
8. Pathmanathan, P., M. S. Shotwell, ..., R. A. Gray. 2015. Uncertainty quantification of fast sodium current steady-state inactivation for multi-scale models of cardiac electrophysiology. *Prog. Biophys. Mol. Biol.* 117:4–18.
9. Beattie, K. A., A. P. Hill, ..., G. R. Mirams. 2018. Sinusoidal voltage protocols for rapid characterisation of ion channel kinetics. *J. Physiol.* 596:1813–1828.
10. Hodgkin, A. L., and A. F. Huxley. 1952. A quantitative description of membrane current and its application to conduction and excitation in nerve. *J. Physiol.* 117:500–544.
11. Vandenberg, J. I., A. Varghese, ..., C. L. Huang. 2006. Temperature dependence of human ether-a-go-go-related gene K+ currents. *Am. J. Physiol. Cell Physiol.* 291:C165–C175.
12. Hobbs, K. H., and S. L. Hooper. 2008. Using complicated, wide dynamic range driving to develop models of single neurons in single recording sessions. *J. Neurophysiol.* 99:1871–1883.
13. Fink, M., and D. Noble. 2009. Markov models for ion channels: versatility versus identifiability and speed. *Philos. Trans. A Math. Phys. Eng. Sci.* 367:2161–2179.
14. Kirsch, G. E., E. S. Trepakova, ..., A. M. Brown. 2004. Variability in the measurement of hERG potassium channel inhibition: effects of temperature and stimulus pattern. *J. Pharmacol. Toxicol. Methods.* 50:93–101.
15. Mirams, G. R., P. Pathmanathan, ..., R. H. Clayton. 2016. Uncertainty and variability in computational and mathematical models of cardiac physiology. *J. Physiol.* 594:6833–6847.
16. Zeng, J., K. R. Laurita, ..., Y. Rudy. 1995. Two components of the delayed rectifier K+ current in ventricular myocytes of the Guinea pig type. Theoretical formulation and their role in repolarization. *Circ. Res.* 77:140–152.
17. ten Tusscher, K. H., D. Noble, ..., A. V. Panfilov. 2004. A model for human ventricular tissue. *Am. J. Physiol. Heart Circ. Physiol.* 286:H1573–H1589.
18. Clerx, M., P. Collins, ..., P. G. Volders. 2016. Myokit: a simple interface to cardiac cellular electrophysiology. *Prog. Biophys. Mol. Biol.* 120:100–114.

19. Hindmarsh, A. C., P. N. Brown, …, C. S. Woodward. 2005. SUNDIALS: suite of nonlinear and differential/algebraic equation solvers. *ACM Trans. Math. Softw.* 31:363–396.

20. Hansen, N. 2006. The CMA Evolution Strategy: A Comparing Review. *In* Toward a New Evolutionary Computation. J. A. Lozano, P. Larrañaga, and I. Inza…, eds. Springer, pp. 75–102.

21. Jasra, A., D. A. Stephens, and C. C. Holmes. 2007. On population-based simulation for static inference. *Stat. Comput.* 17:263–279.

22. Haario, H., E. Saksman, and J. Tamminen. 2001. An adaptive Metropolis algorithm. *Bernoulli.* 7:223–242.

23. Clerx, M., M. Robinson, …, D. J. Gavaghan. 2019. Probabilistic inference on noisy time series (PINTS). *Journal of Open Research Software.* 7:23.

24. Gilks, W. R., N. Best, and K. Tan. 1995. Adaptive rejection Metropolis sampling within Gibbs sampling. *Appl. Stat.* 44:455–472.

25. Ishii, K., M. Nagai, …, M. Endoh. 2003. Dissociation of E-4031 from the HERG channel caused by mutations of an amino acid results in greater block at high stimulation frequency. *Cardiovasc. Res.* 57:651–659.

26. Fermini, B., J. C. Hancox, …, J. I. Vandenberg. 2016. A new perspective in the field of cardiac safety testing through the comprehensive in vitro proarrhythmia assay paradigm. *J. Biomol. Screen.* 21:1–11.

27. Chang, K. C., S. Dutta, …, Z. Li. 2017. Uncertainty quantification reveals the importance of data variability and experimental design considerations for in silico proarrhythmia risk assessment. *Front. Physiol.* 8:917.

28. Lei, C. L., K. Wang, …, L. Polonchuk. 2017. Tailoring mathematical models to stem-cell derived cardiomyocyte lines can improve predictions of drug-induced changes to their electrophysiology. *Front. Physiol.* 8:986.

29. Lei, C. L., M. Clerx, …, G. R. Mirams. 2019. Rapid characterisation of hERG potassium channel kinetics II: temperature dependence. *Biophys. J*:117:XXX.

Biophysical Journal, Volume *117*

Supplemental Information

Rapid Characterization of hERG Channel Kinetics I: Using an Automated High-Throughput System

Chon Lok Lei, Michael Clerx, David J. Gavaghan, Liudmila Polonchuk, Gary R. Mirams, and Ken Wang

Contents

S1 Details of voltage clamp protocol 1
S2 Ramps in the staircase protocol 2
S3 Electrophysiology solutions 2
S4 Recording techniques 3
S5 Automated quality control 4
S6 Synthetic data studies 7
S7 Sweeps comparison 15
S8 Posterior predictive quantification 16
S9 Remaining relative root mean square error (RRMSE) histograms 19
S10 Practical identifiability of model parameters 19
S11 Mean model parameters 22
S12 Estimated voltage error and other quality control parameters 22
S13 Estimated voltage error and parameter variability 23
References 25

S1 Details of voltage clamp protocol

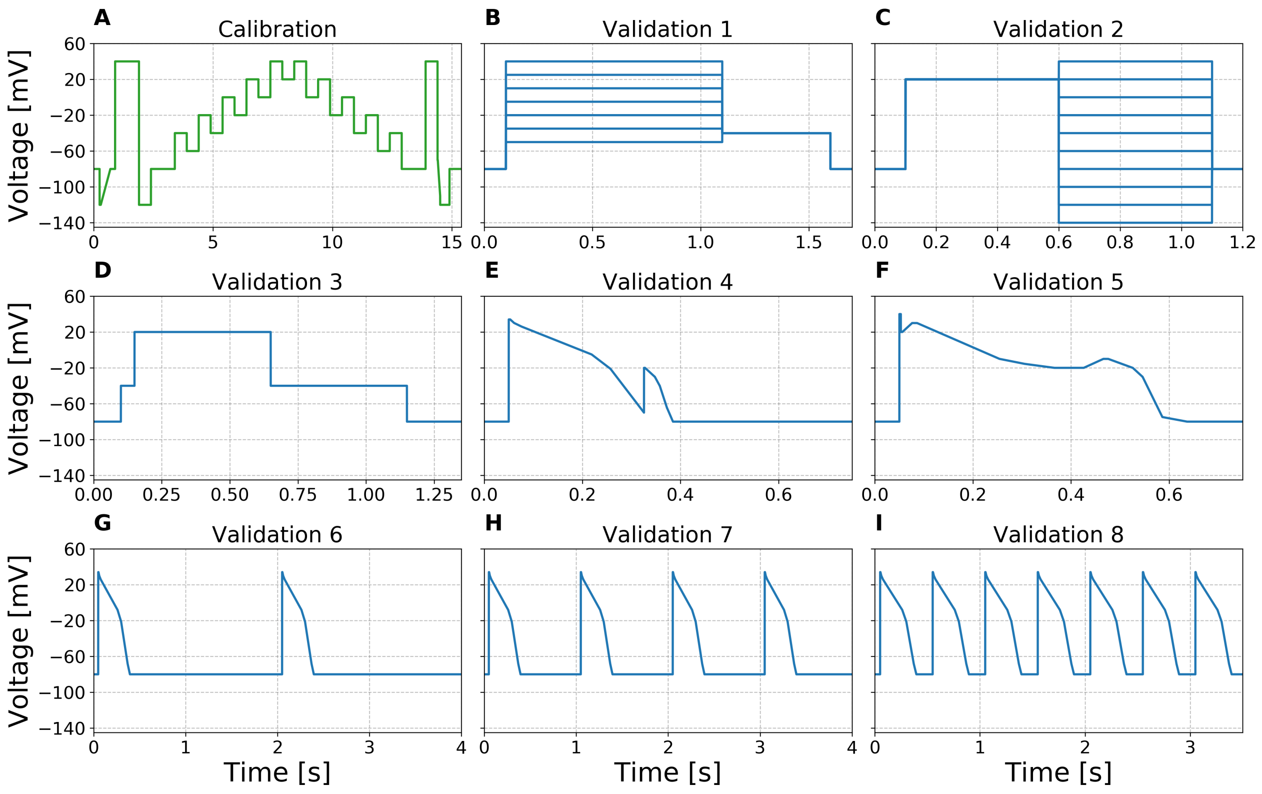

Figure S1. All voltage clamp protocols used in the study, from **A** to **I** are (green) our newly developed staircase protocol, (blue) the activation current-voltage (I-V) protocol, the steady-state inactivation I-V protocol, the hERG screening protocol, the delayed afterdepolarization (DAD)-like protocol, the early afterdepolarization (EAD)-like protocol, and the action potential (AP)-like protocol with beating frequency 0.5 Hz, 1 Hz and 2 Hz. All protocols are shown with the same voltage axes for comparison; however due to different time scale, each of them has its own time axis.

S1.1 Calibration: Staircase protocol

The full protocol is comprised of a 250 ms step at holding potential of −80 mV, followed by a 50 ms 'leak step' at −120 mV, and a 400 ms 'leak ramp' from −120 mV to −80 mV, before a 200 ms back at holding potential. This was followed by a 1 s 'activation step' at 40 mV and a 500 ms 'closing step' at −120 mV, before returning to holding potential for 1 s. Then the 9.5 s staircase portion of the protocol (the details is described below), before a return to holding potential for 500 ms. Finally, it was followed by a reversal potential estimation portion which is composed of a 500 ms step to 40 mV, and a 10 ms step to −70 mV to remove capacitance effect, then followed by a 100 ms 'reversal potential ramp' starting from −70 mV to −110 mV, before a 390 ms step to −120 mV, and return to holding potential for 500 ms.

The staircase portion of the protocol consists of a range of 500 ms steps up and down as discussed in main text. It is comprised of two sets of steps, the first set alternates between $V_{step,1}$ and $V_{step,2}$, each for 500 ms. There are 5 different $V_{step,1}$ and $V_{step,2}$; $V_{step,1}$ ranged from −40 mV to 40 mV, and $V_{step,2}$ ranged from −60 mV to 20 mV, both in 20 mV increments. The second set alternates between $V_{step,3}$ and $V_{step,4}$, each for 500 ms. There are 5 different $V_{step,3}$ and $V_{step,4}$; $V_{step,3}$ ranged from 40 mV to −40 mV, and $V_{step,4}$ ranged from 0 mV to −80 mV, both in 20 mV decrements.

This protocol is shown in Figure S1A. A time series version of the full protocol is available at https://github.com/CardiacModelling/hERGRapidCharacterisation/blob/master/protocol-time-series/protocol-staircaseramp.csv.

S1.2 Validation 1: Activation I-V protocol

From the initial period 100 ms at holding potential of −80 mV, a step to V_{step} for 1 s, followed by a 500 ms step to −40 mV, before a 100 ms step back to holding potential; this was repeated 7 times with a different V_{step} on each repeat. V_{step} ranged from −50 mV to 40 mV in 15 mV increments. This protocol is shown in Figure S1B.

S1.3 Validation 2: Steady-state inactivation I-V protocol

From the initial period 100 ms at holding potential of −80 mV, a step to 20 mV for 500 ms, followed by a step to V_{step} for 500 ms, before a 100 ms step back to holding potential; this was repeated 10 times with a different V_{step} on each repeat. V_{step} ranged from −140 mV to 40 mV in 20 mV increments. This protocol is depicted in Figure S1C.

S1.4 Validation 3: hERG screening protocol

From the initial period 100 ms at holding potential of −80 mV, a step to −40 mV for 50 ms, and a step to 20 mV for 500 ms, followed by a step to −40 mV for 500 ms, before a 200 ms step back to holding potential. This protocol is shown in Figure S1D.

S1.5 Validation 4-8: DAD-like, EAD-like, APs-like protocols

Details are described in Table S1, and each protocol is shown in Figure S1E-I respectively.

S2 Ramps in the staircase protocol

As discussed in the main text, protocol design, the two ramps implemented in the staircase protocol are designed to estimate the leak current and to experimentally estimate the E_K value. Figure S2 shows an example of using the two ramps to estimate the leak current and the E_K value. The top three panels show the staircase voltage clamp protocol (grey), an example of raw currents before (blue) and after (orange) E-4031 application, and the corresponding estimated I_{Kr} (green; the difference between the blue and orange traces), respectively. The greyed out sections highlight the two ramps in the staircase protocol. Bottom left shows the I-V curves of the two raw currents measured under the first ramp. Linear regressions were applied, and the results are shown as dashed lines, where the fitted slope and y-interception point were used to estimate the leak current parameters (Eq. 12 in the main text). Bottom right shows the I-V curve of the leak-corrected, E-4031 subtracted I_{Kr} measured under the second ramp. A third order polynomial regression was applied, and the result is shown as dashed line. The E_K value was then estimated as the x-interception point, shown as red vertical line.

S3 Electrophysiology solutions

The compositions of all the electrophysiology solutions, including both the external solutions (bath solutions) and the internal solution (equivalent to the pipette solution in manual patch clamp), are shown in Table S2. External solutions were added in the following order: first 'fill chip' solution to the measurement chip, and the suspended hERG cells, then the 'seal enhancer' solution for enhancing the seal by forming CaF crystal around the cells (note they have extra high concentration of Ca^{+}, so we need to reduce/dilute it later), followed by adding the extracellular 'reference' solution for Ca^{+} dilution. All the voltage clamp measurements were performed after adding all these external solutions.

The solutions were added sequentially to the wells, by removing half of the previous solutions from the wells each time. Therefore, the final ratios of the external (extracellular) solution are 1:1:2 — proportions of 0.25 of the 'Fill Chip' concentrations,

DAD-like protocol		
**Type**	**V [mV]**	**Duration [ms]**
Step	-80	50
Step	34	3
Ramp	30	8
Ramp	26	15.2
Ramp	-5	142.6
Ramp	-21	38.4
Ramp	-70	68.6
Step	-20	2
Ramp	-30	20
Ramp	-40	10
Ramp	-65	15
Ramp	-80	12
Step	-80	15.2
Step	-80	350

EAD-like protocol		
**Type**	**V [mV]**	**Duration [ms]**
Step	-80	50
Step	40	3
Step	20	3
Ramp	30	20
Step	30	10
Ramp	-10	168
Ramp	-15.5	50.6
Ramp	-20	61.2
Step	-20	60
Ramp	-10	40
Step	-10	10
Ramp	-20	50
Ramp	-30	20
Ramp	-75	40.5
Ramp	-80	50
Step	-80	13.7
Step	-80	100

Single AP-like protocol		
**Type**	**V [mV]**	**Duration [ms]**
Step	-80	50
Step	34	3
Ramp	30	8
Ramp	26	15.2
Ramp	-8	183.6
Ramp	-21	39
Ramp	-68	70.5
Ramp	-80	25.2
Step	-80	—

Table S1. Details of the DAD-like (validation 4), EAD-like (validation 5), APs-like (validation 6-8) protocols. It shows as a sequence of steps and ramps that approximates different types of action potential shapes, as these are the only available settings in the automated machine used. The voltage (V) in the type Ramp represents the final targeted voltage that the ramp finishes, starting from the previous voltage within the given duration; for example, the first ramp in the EAD-like protocol means it starts from 34 mV and ramps to 30 mV in 8 ms. The single AP-like protocol shows the protocol for one unit AP-like protocol that repeats in 0.5 Hz, 1 Hz, and 2 Hz.

0.25 of the 'Seal Enhancer' concentrations, and 0.5 of the 'Reference' concentrations, as shown in the 'Final Extracellular' solution in Table S2. For each well, the volume of the final solution during recording is 80 µl.

**Solution**<br>**pH value (titrated with)**<br>**Osmolarity [mOsm]**		*Intracellular*<br>*pH 7.2 (KOH)*<br>*260-300*	*Fill Chip*<br>*pH 7.4 (NaOH)*<br>*300-330*	*Seal Enhancer*<br>*pH 7.4 (HCl)*<br>*290-330*	*Reference*<br>*pH 7.4 (HCl)*<br>*290-330*	*Final Extracellular*
**Chemicals**	**Source / Cat#**	**[ ] in mM**	**[ ] in mM**	**[ ] in mM**	**[ ] in mM**	**[ ] in mM**
NaCl	Merck / K38447104807	10	150	80	80	97.5
KCl	Merck / K36782536	10	4	4	4	4
KF	Acros Organics / 201352500	100	—	—	—	—
$MgCl_2$	Merck / A914133908	—	1	1	1	1
$CaCl_2$	Acros Organics/ 349615000	—	1.2	5	1	2.05
HEPES	Applichem A1069	10	10	10	10	10
Glucose	Fluka / 49159	—	5	5	5	5
NMDG	Fluka 66930	—	—	60	40	35
EGTA	Fluka / 03778	20	—	—	—	—
Sorbitol	Sigma / S1876	—	—	—	40	20

Table S2. Electrophysiology solutions for hERG assay on the Nanion SyncroPatch 384PE machine, all solutions are sterile filtered. All hERG cells were suspended in 1/3 Extracellular Fill Chip Solution + 2/3 Hanks' Balanced Salt Solution (HBSS).

S4 Recording techniques

All experiments were performed with Nanion SyncroPatch 384PE machine with software PatchControl384PE (v. 1.5.6 Build 22) and current traces data were exported using their complementary software DataControl384 (v. 1.5.0 Customer Release). Temperature was controlled by Nanion temperature control unit with software PE384TemperatureControl. The machine comes with a measurement chip consists of 364 wells, with 16 rows by 24 columns.

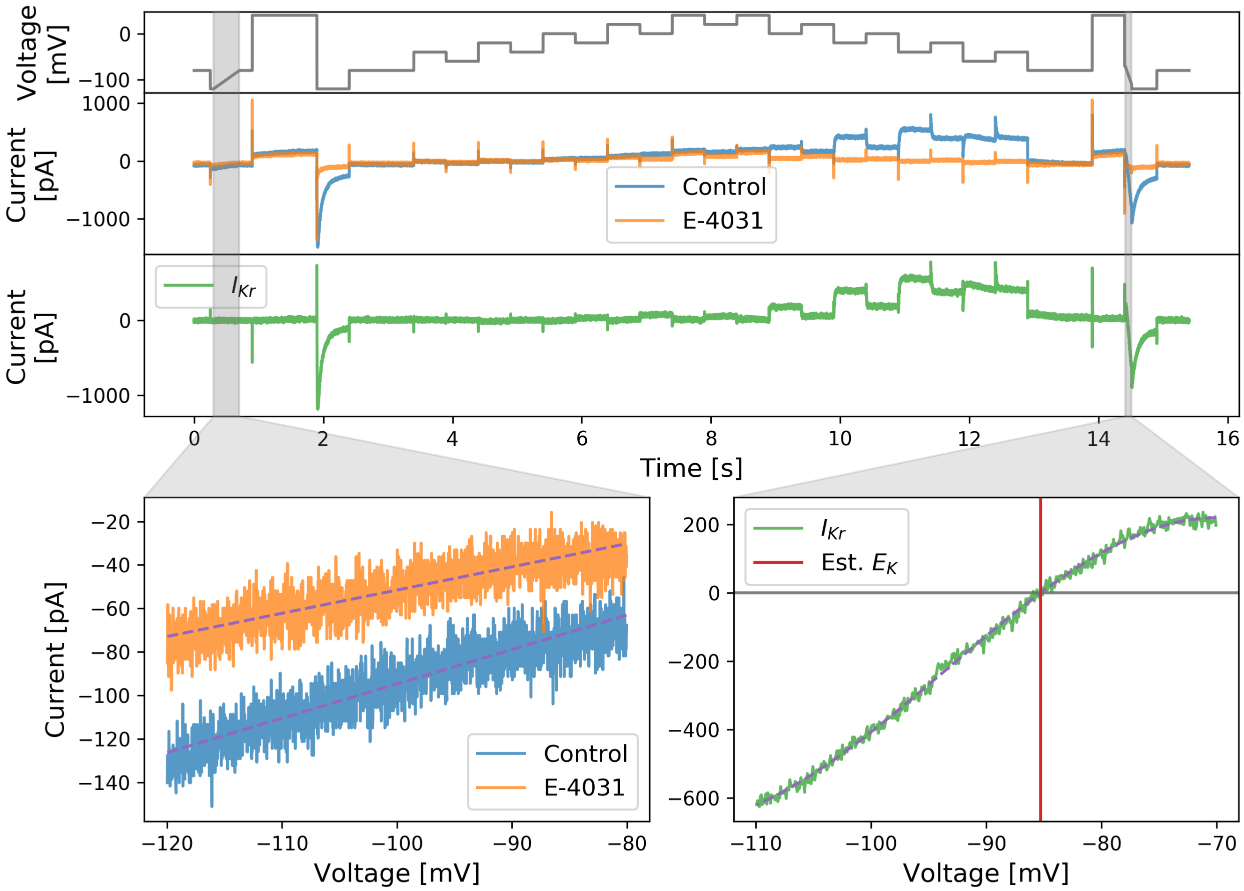

Figure S2. Two ramps (greyed out sections) implemented in the staircase protocols. The first ramp is designed to estimate the leak current; the second ramp is designed to experimentally estimate the E_K value. Top three panels show the staircase voltage clamp protocol (grey), the raw currents before (blue) and after (orange) E-4031 application, and the estimated I_{Kr} (green; the subtraction between the blue and orange traces), respectively. Below shows the I-V curves measured during the two ramps. Linear regressions were applied to each of the I-V relation in the first ramp, shown as dashed lines. Third order Polynomial regression was applied to the I-V curve in the second ramp, shown as dashed lines.

S5 Automated quality control

Here we present a more detailed selection results of our quality control which does not require any manual intervention. The full details of our automated quality control criteria are summarised in Table 1 in the main text. A well must pass all the listed criteria in order to be selected.

In Figure S3, we break down the selection results and show the results of each criterion in our automated quality control. On the left, the bar chart shows the number of wells removed by each quality control criterion. There were 22 ‘no cell’ wells, where the platform decided there was no valid estimation of R_{seal}, C_m, and R_{series} and it was likely that no cell was clamped in these wells. Our three **QC1** criteria are used as part of the automated high-throughput machine quality control, which can eliminate up to 46 wells out of the 201 wells that we manually decided to remove. We then added the other criteria to improve the selection process, which allow us to eliminate a total of 173 wells, and achieved a positive predictive value of >86 %. On the right, we show the number of wells commonly removed by any pair of criteria. This shows that most of our criteria are quite independent, and are assessing different features of the recordings.

We note that our automated quality control can achieve a positive predictive value of >86 %. In Figure S4, we show 6 typical examples of the ‘bad recordings’ that we manually removed. The manually removed bad recordings are compared against the good recordings. Top panel shows our staircase protocol. Then we show 3 good recordings (green) and 6 manually removed bad recordings (orange/red). We found our manually removed recordings fall into two main categories, as coloured, orange and red. For the first category (orange), although they seem to contain I_{Kr}, they are heavily ‘contaminated’ by other signals which are most probably a combination of leak and endogenous currents. For the second category (red), the recordings lack any

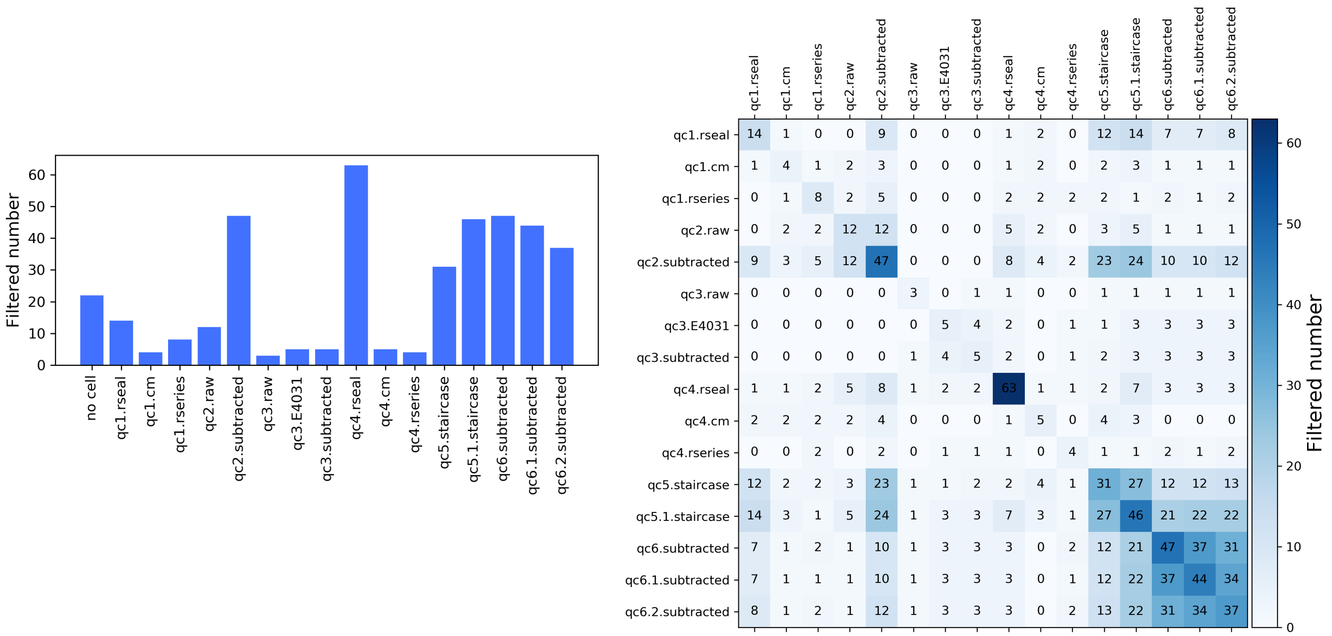

Figure S3. Selection results of each criterion from our automated quality control. **(Left.)** Showing the number of wells filtered out by each quality control criterion as bar chart. **(Right.)** Showing the number of wells filtered out by both the row and column criteria. The automated high-throughput machine also has some simple quality control implemented, which are our three **QC1** criteria.

characteristic dynamics of I_{Kr}, for example during the first big repolarising step from 40 mV to −120 mV, the recordings do not show any negative spikes that we would associate with hERG opening. Therefore, none of them are considered as good recordings of I_{Kr}.

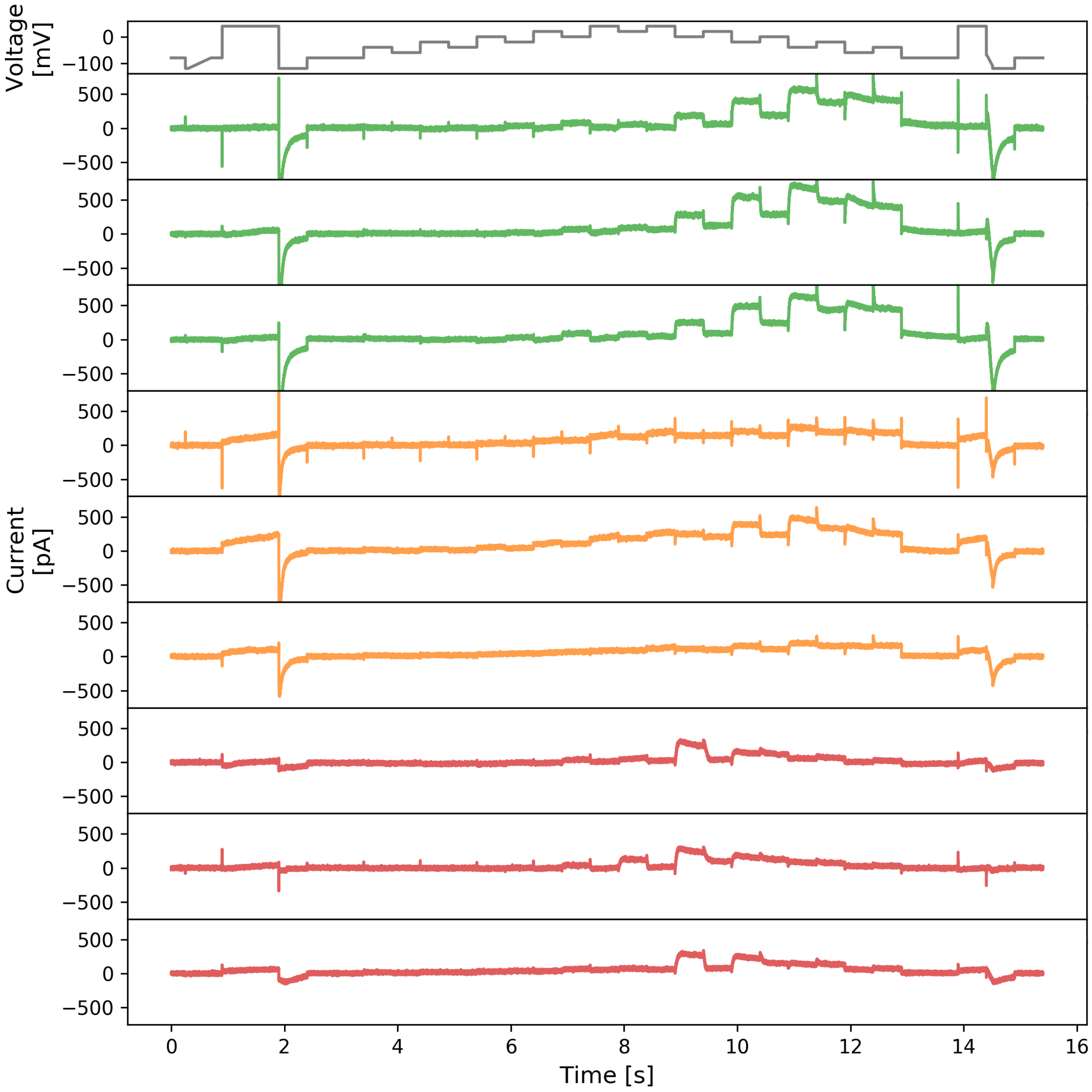

Figure S4. A comparison of the good recordings and our manually removed bad recordings. Top panel shows our staircase protocol. Following are 3 good recordings (green) and 6 manually removed bad recordings (orange/red). We found our manually removed recordings fall into two main categories, as coloured, orange and red.

S6 Synthetic data studies

S6.1 Introduction

We perform a synthetic data study prior to implementing the actual experiments for two reasons: design of protocols and test of analysis.

First, given the mathematical model of the hERG channel, we are able to deduce what would be the best protocols to tease out the kinetics of the underlying model. This can alleviate a common issue, identifiability when undertaking model fitting. Because typically one constructs the problem into inverse problems when trying to parameterise mathematical models, however the identifiability issue arises because of the poorly informed experimental data. Therefore we utilise a synthetic study to design and optimise protocols to have sufficient information for rapid characterisation.

Second, we are able to test our analysis technique, to ascertain whether it is robust enough for our purpose — to recover the parameters of the model given the data. Most of our ion channel models can be written as

$$I = f(V,t;\boldsymbol{\theta},I_0), \tag{S1}$$

where I is the current (output of the model, the observable in experiments), V is the voltage, and $\boldsymbol{\theta}$ is the vector of parameters within the model. The models are usually formulated as differential equations which therefore requires initial conditions I_0. The dependency on initial conditions I_0 can usually be eliminated by running the model long enough to reach a (pseudo-)steady state. Then with our analysis techniques, given the output I with inputs V and t, we aim to infer the values of the parameters $\boldsymbol{\theta}$, hence the overall process is termed an inverse problem. Therefore, we generate synthetic data (with added synthetic noise) with some 'true' parameters $\boldsymbol{\theta}^{\text{true}}$, and we ask, how confident are we in our inferred parameters?

S6.2 Methods

S6.2.1 Generating synthetic data

We generate synthetic data by simulating the current I, with some fixed known parameter sets $\{\boldsymbol{\theta}^{\text{true}}\}$, voltage protocol $V_{prt}(t)$, initial values I_0, and sampling time (time-step) Δt.

First, the choice of $\{\boldsymbol{\theta}^{\text{true}}\}$ could be arbitrary, but we used the parameters identified from a previous study[1] (Table F11 Cell #5), $\boldsymbol{\theta}^{\text{lit}}$, to utilise prior knowledge. We generated $\{\boldsymbol{\theta}^{\text{true}}\} = \{\boldsymbol{\theta}^{\text{true},1}, \boldsymbol{\theta}^{\text{true},2}, \cdots, \boldsymbol{\theta}^{\text{true},N_e}\}$ with each $\boldsymbol{\theta}^{\text{true},j} = (\theta_1^{\text{true},j}, \theta_2^{\text{true},j}, \cdots, \theta_N^{\text{true},j})^T$ sampled from

$$\theta_i^{\text{true},j} \sim \mathcal{N}(\theta_i^{\text{lit}}, \rho_i^2), \tag{S2}$$

where $i = 1,2,\cdots,N$ for N parameters in the model, and $(\cdots)^T$ represents the transpose. $\mathcal{N}$ denotes the normal distribution and ρ^2 is the variance for which we chose a value of $\rho_i = 0.2|\boldsymbol{\theta}_i^{\text{lit}}|$. That is, we assume that we performed N_e experiments (recordings), and there exists variability between experiments. Assuming each experiment was performed identically, then the variability that we are simulating is *cell-to-cell variability*.

We can take the notion of variability further, by removing the assumption of independence *between* model parameters. We assume there exists an underlying correlation between each model parameter, which can be described by a covariance matrix Σ. Therefore we can rewrite the underlying distribution of the parameters as being taken from a (covarying) multivariate normal distribution, that is

$$\boldsymbol{\theta}^{\text{true},j} \sim \mathcal{N}(\boldsymbol{\theta}^{\text{lit}}, \boldsymbol{\Sigma}). \tag{S3}$$

The correlation between parameters using the correlation matrix is then defined as

$$\text{corr}(\boldsymbol{\theta}) = \text{diag}(\boldsymbol{\Sigma})^{-1/2}\ \boldsymbol{\Sigma}\ \text{diag}(\boldsymbol{\Sigma})^{-1/2}, \tag{S4}$$

where $\text{diag}(\cdot)$ denotes the matrix of the diagonal entries and its (i,i) entry is chosen to be ρ_i^2. We randomly generated the correlation matrix that satisfies the positive semi-definite condition for this synthetic data study.

Second, we fix the voltage V of the model at $V_{prt}(t)$, which is the staircase protocol that we developed for the high-throughput systems. Third, for the initial values I_0, we ran the model at $V = -80$ mV for a long period (100 s), to allow the model to settle at its steady state at $V = -80$ mV. Since we are able to mimic this in the actual experiments, we assume the model does not depend on the choice of I_0, that is $I \approx f(V,t;\boldsymbol{\theta})$.

Finally, we add synthetic noise which follows a normal distribution with a mean of zero and standard deviation σ (i.e. $\sim \mathcal{N}(0,\sigma^2)$) to the simulated traces with $\Delta t = 0.5$ ms. We chose σ at a reasonable scale, $\sigma = 11$ pA, to mimic the high frequency noise observed from some of our pilot experiments using the high-throughput system.

S6.2.2 Inferring parameters

To infer the parameters, we use a two-step approach. Firstly, we use a global optimisation algorithm, CMA-ES[2], to identify the parameters. Secondly, we run Markov-chain Monte Carlo (MCMC) to explore and quantify the uncertainty of the identified parameters.

In the CMA-ES optimisation, we used the sum of squares error measure of the whole trace as our objective function. To alleviate any potential issues arising due to a constrained objective function, we applied a transformation g that maps the positively constrained model parameters $\{\theta_i\}$, with $\theta_i \in [0,\infty]$, to $\{\phi_i\} \in \mathbb{R}^N$, an unconstrained search space for optimisation, which is simply a log-scale transformation:

$$\theta_i = g^{-1}(\phi_i) = e^{\phi_i}. \tag{S5}$$

We then further considered the physical constraints for the rate constants in the kinetics parameters[1], which has the form $k = A\exp(BV)$. For parameters of the form A, $[\theta_i^{\min}, \theta_i^{\max}]$ is chosen to be $[10^{-7}, 10^3]\,\mathrm{ms}^{-1}$; and for parameters of the form B, $[\theta_i^{\min}, \theta_i^{\max}]$ is chosen to be $[10^{-7}, 0.4]\,\mathrm{mV}^{-1}$.

For the MCMC, we used a population MCMC[3] algorithm with adaptive Metropolis[4] algorithm as the base sampler. The starting point of the population MCMC was chosen to be the CMA-ES inferred parameters. As a good practice, the population MCMC was repeated 3 times to ensure the convergence of the MCMC chains. We chose the posterior measure to be

$$p(\boldsymbol{\phi},\sigma|\mathbf{y}) = \frac{p(\boldsymbol{\phi})p(\mathbf{y}|\boldsymbol{\phi},\sigma)}{p(\mathbf{y})} \propto p(\boldsymbol{\phi})p(\mathbf{y}|\boldsymbol{\phi},\sigma), \tag{S6}$$

$$p(\boldsymbol{\phi}) \sim \mathcal{U}(\boldsymbol{\phi}^{\min}, \boldsymbol{\phi}^{\max}), \tag{S7}$$

$$p(\mathbf{y}|\boldsymbol{\phi},\sigma) = \frac{1}{\sqrt{2\pi\sigma^2}} \exp\left(-\sum_k \frac{\left(f(V_{prt}, t_k; g^{-1}(\boldsymbol{\phi})) - \mathbf{y}|_{t_k} \right)^2}{2\sigma^2} \right). \tag{S8}$$

Here, $\mathbf{y}$ is the data and $\mathbf{y}|_{t_k}$ denotes the data at time t_k. The likelihood, $p(x|\boldsymbol{\phi})$, in Eq. S8 is the Gaussian noise version of the sum of square difference measure used in the CMA-ES.

S6.2.3 Hierarchical Bayesian model

In order to infer the correlation *between* model parameters, $\mathrm{corr}(\boldsymbol{\theta})$ in Eq. S4, the mean, and the variability between cells, we used a multi-level modelling technique which works under the Bayesian framework, known as a hierarchical Bayesian model. This allows us to combine all the results from each individually performed experiment to inform the prediction of future experiments.

A schematic of our hierarchical Bayesian model structure is shown in Figure S5. The full hierarchical Bayesian model is

$$\begin{aligned}\mathcal{L}\left(\boldsymbol{\mu}, \boldsymbol{\Sigma}, \left\{\boldsymbol{\phi}_j, \sigma_j\right\}_{j=1}^{N_e} | \left\{\mathbf{y}_j\right\}_{j=1}^{N_e}\right) \propto & \prod_{j=1}^{N_e} p\left(\mathbf{y}_j|\boldsymbol{\phi}_j,\sigma_j\right) \\ & \times p\left(\left\{\boldsymbol{\phi}_j,\sigma_j\right\}_{j=1}^{N_e} |\boldsymbol{\mu},\boldsymbol{\Sigma}\right) \\ & \times p(\boldsymbol{\mu},\boldsymbol{\Sigma}) \times \prod_{j=1}^{N_e} p\left(\sigma_j\right),\end{aligned} \tag{S9}$$

where all symbols have their usual meaning as defined above, $\mathcal{L}$ is the full posterior, and $\boldsymbol{\mu}, \boldsymbol{\Sigma}$ are the hyperparameters of the hierarchical model which are the means and covariance matrix of the model parameters. We assume the model parameters follow a multivariate log-normal distribution, thus the hyperparameters define the mean and covariance matrix of this distribution. The three terms in Eq. S9 are: 1. the likelihood of *all* the individual (low-level) experiments; 2. the likelihood of the hyperparameters; and 3. the priors of the hyperparameters (also known as 'hyper-priors') and the prior of σ_j which we do not infer its hyperparameters.

For computational ease, we chose the prior of the hyperparameters to be a multivariate normal distribution for the $\boldsymbol{\mu}$ and an inverse-Wishart distribution $\mathcal{W}^{-1}$ for the $\boldsymbol{\Sigma}$, which is the respective conjugate prior. Suppose N_e individual parameters $\{\boldsymbol{\theta}_j\}_{j=1}^{N_e}$ have been observed, then we have

$$\left\{\ln\boldsymbol{\theta}_j\right\}_{j=1}^{N_e} \sim \mathcal{N}(\boldsymbol{\mu},\boldsymbol{\Sigma}), \tag{S10}$$

and with the conjugate prior

$$p(\boldsymbol{\mu},\boldsymbol{\Sigma}) = p(\boldsymbol{\mu}|\boldsymbol{\Sigma})p(\boldsymbol{\Sigma}), \tag{S11}$$

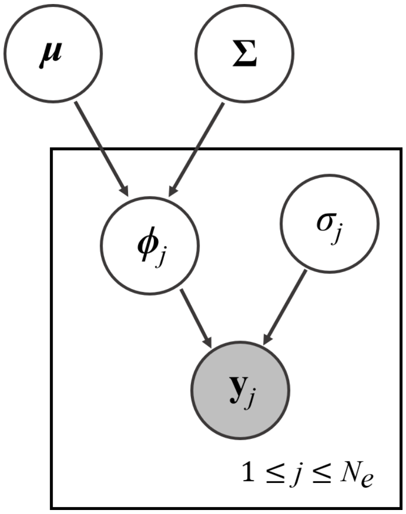

Figure S5. Hierarchical Bayesian model showing parameter dependency for combining multiple experiments. $\boldsymbol{\mu}, \boldsymbol{\Sigma}$ are the hyperparameters of the hierarchical model which represent the mean and covariance matrix, respectively, of the individual 'low-level' parameters, $\{\boldsymbol{\theta}_j, \sigma_j\}_{j=1}^{N_e}$ are the set of individual 'low-level' parameters for each of the N_e measurements in the high-throughput experimental recordings $\{\mathbf{y}_j\}_{j=1}^{N_e}$. The parameters in the box repeat for multiple wells and are indexed as the jth experiment (or dataset). All parameters, and their probability distributions, are inferred from the shaded variable $\mathbf{y}_j$, the experimental data. Prior distributions are required for the parameters with no inward-pointing arrows.

where

$$p(\boldsymbol{\mu}|\boldsymbol{\Sigma}) \sim \mathcal{N}\left(\boldsymbol{\mu}_0, \frac{1}{m}\boldsymbol{\Sigma}\right), \quad \text{and} \quad p(\boldsymbol{\Sigma}) \sim \mathcal{W}^{-1}(\boldsymbol{\Psi}, \nu). \tag{S12}$$

$\boldsymbol{\mu}_0, m, \boldsymbol{\Psi}, \nu$ are the prior parameters, where m, ν are respectively the strength of the prior mean $\boldsymbol{\mu}_0$ and $\boldsymbol{\Psi}$ which determines the prior of the covariance $\boldsymbol{\Sigma}$. Then the posterior distribution of the hyperparameters becomes

$$p\left(\boldsymbol{\mu} \,|\, \boldsymbol{\Sigma}, \left\{\ln\boldsymbol{\theta}_j\right\}_{j=1}^{N_e}\right) \sim \mathcal{N}\left(\frac{N_e\bar{\boldsymbol{\theta}} + m\boldsymbol{\mu}_0}{n+m}, \frac{1}{m+N_e}\boldsymbol{\Sigma}\right), \text{ and} \tag{S13}$$

$$p\left(\boldsymbol{\Sigma} \,|\, \left\{\ln\boldsymbol{\theta}_j\right\}_{j=1}^{N_e}\right) \sim \mathcal{W}^{-1}\left(\boldsymbol{\Psi} + N_e\boldsymbol{S} + \frac{N_e m}{N_e+m}(\bar{\boldsymbol{\theta}} - \boldsymbol{\mu}_0)(\bar{\boldsymbol{\theta}} - \boldsymbol{\mu}_0)^T, N_e + \nu\right), \tag{S14}$$

where

$$\bar{\boldsymbol{\theta}} = \frac{1}{N_e}\sum_{j=1}^{N_e} \ln\boldsymbol{\theta}_j, \text{ and} \tag{S15}$$

$$\boldsymbol{S} = \frac{1}{N_e}\sum_{j=1}^{N_e} (\bar{\boldsymbol{\theta}} - \ln\boldsymbol{\theta}_j)(\bar{\boldsymbol{\theta}} - \ln\boldsymbol{\theta}_j)^T. \tag{S16}$$

We use the Metropolis within Gibbs (MwG)[5] sampling method to explore the full hierarchical Bayesian model. The number of parameters we have in Eq. S9 is $N(N+1)/2 + (N_e+1)N + N_e$. For our choice of hERG model and the size of the dataset, we are expecting $N = 9$ and $N_e > 100$. This gives us more than 1000 parameters for which we wish to infer probability distributions. It is computationally expensive and infeasible to use other standard algorithms, such as the population MCMC, and even MwG can be very time consuming. We therefore further simplify the MwG to approximate the full posterior sampling, which we have termed '*pseudo-MwG*'. We confirm that the pseudo-MwG can approximate the MwG very well in the results below.

Under our pseudo-MwG, we assume that the likelihoods of our individual experiments are unlikely to be affected by the top-level distribution, due to our information-rich staircase protocol having thousands of data points rather than the ~ 100 wells. We therefore separate the sampling steps between the likelihood of all the individual experiments and the likelihood of the hyperparameters. That is, we first *independently* sample the likelihood of each individual experiment, using population

MCMC alogrithm. Then we sample the hyperparameters using Eq. S10–S16, where $\left\{\ln \boldsymbol{\theta}_j\right\}_{j=1}^{N_e}$ become $\left\{\ln \boldsymbol{\theta}_{j,l}\right\}_{j=1}^{N_e}$ which are the independently obtained l^{th} samples of the individual experiments j. Note that this is only valid when the individual experiments are far more information-rich than the number of repeats. We check these assumptions in the results below.

To obtain the posterior predictive distribution $p(\boldsymbol{\theta}|\cdots)$ which allows us to make prediction about how the future experiments would behave, where $(\cdots)$ indicates all other variables appear in Eq. S9, we use

$$p(\boldsymbol{\theta}|\cdots) = \int_{\boldsymbol{\Theta}} p(\boldsymbol{\theta}|\boldsymbol{\Theta})p(\boldsymbol{\Theta}|\cdots)\, \mathrm{d}\boldsymbol{\theta}, \tag{S17}$$

where $\boldsymbol{\Theta} = (\boldsymbol{\mu}, \boldsymbol{\Sigma})^T$. This can be approximated by summing over the probability density functions which are defined by the samples of $\boldsymbol{\Theta}$.

S6.3 Results/Discussion

S6.3.1 Single synthetic experiment

We start by showing the staircase protocol is information-rich enough to identify the 'true' parameter set in a synthetic data study using our protocol. Figure S6 shows the results of inferring model parameters on a synthetic experiment, where $\boldsymbol{\theta}^{\text{true}} = \boldsymbol{\theta}^{\text{lit}}$ obtained from a previous study[1] (Table F11 Cell #5). It shows the three independently sampled marginal posterior distributions of each parameter (first and third columns), with indications of the 'true' parameters $\boldsymbol{\theta}^{\text{true}}$ (black dashed lines) which we used to generate the synthetic data, and the CMA-ES inferred parameters (red lines). Both the traces (second and fourth columns) and the three independently run posterior distributions show a good indication of the convergence of the MCMC chains. We are able to recover the 'true' parameters $\boldsymbol{\theta}^{\text{true}}$ with high accuracy and a narrow credible interval using our inference techniques together with our developed staircase protocol. Therefore we are confident that, with both the high information-content protocol and the inference techniques, it is theoretically possible to infer all parameters of the model.

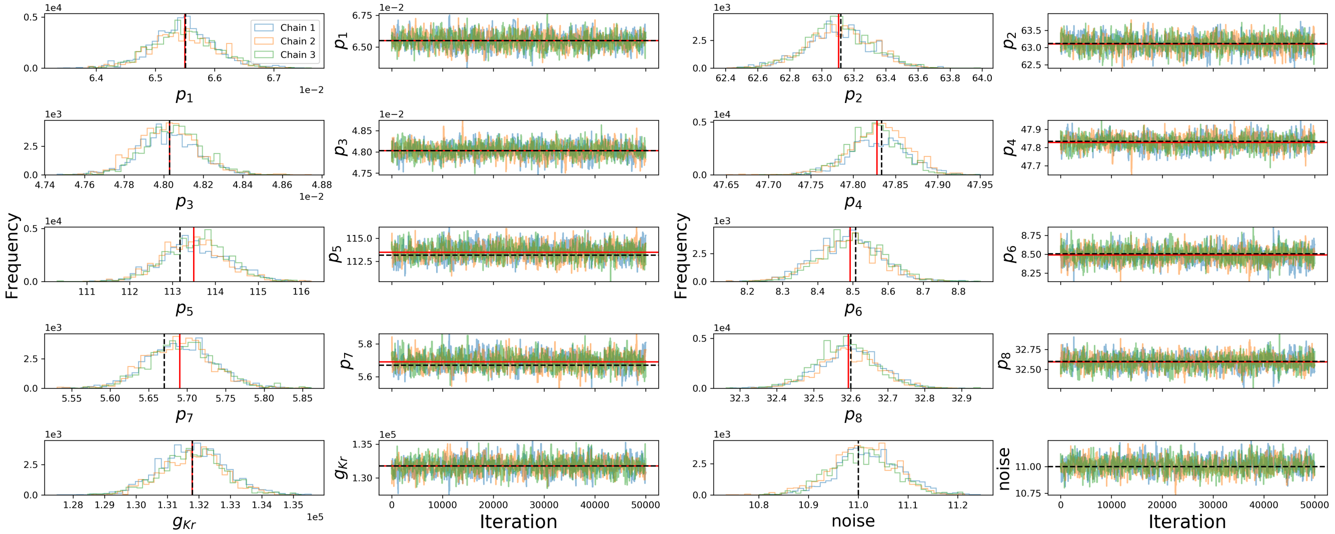

Figure S6. Parameter inference of single synthetic experiment, $N_e = 1$. **First, Third columns:** Show the marginal histograms of the posterior distribution of each parameter. **Second, Fourth columns:** The trace plots for our MCMC chains indicating that our MCMC chains have converged. Each panel shows the posterior distribution of 3 independently run MCMC, and their extremely good agreement assures the chains are well mixed. The true (synthetic) parameters are indicated as black dashed lines and the CMA-ES inferred parameters are shown as red lines.

S6.3.2 Hierarchical synthetic experiments

Figure S7 shows the results of the synthetic data study using hierarchical Bayesian model with $N_e = 120$. It shows the marginal histograms of the model parameters for each individual experiment (left y-axis) and the marginal posterior predictive distribution (right y-axis, red lines). This synthetic data study is equivalent to have N_e repeats of the same experiment. Unlike the single experiment study above, the implications of the obtained posterior predictive distribution $p(\boldsymbol{\theta}|\cdots)$ are much more powerful and can be viewed in two ways.

First, we can see this as the underlying distribution that governs the parameters. That is, with this, we can try to understand – through the model – what the hERG channel is doing in the cells. To do so, we compare it with the 'true' underlying distribution

of parameters (black dashed lines), i.e. the multivariate normal distribution in Eq. S3. The marginal posterior predictive distributions closely resemble the 'true' distribution. This indicates that we are able to recover the underlying distribution of the parameters with high accuracy too, and therefore we can rely on it to study the behaviour of hERG cells in actual experiments.

Second, as the name implies, this is a *predictive* distribution. That is, given the observed individual experiments, we infer a distribution which allows us to predict what might happen in a future experiment. To do this, we can view the posterior predictive distribution in Eq. S17 as $p(\boldsymbol{\theta}_{N_e+1}|\cdots)$, where $\boldsymbol{\theta}_{N_e+1}$ is our 'future' $(N_e+1)^{\text{th}}$ experiment that we perform. Therefore the distribution that we construct is able to tell us what is likely to happen in the future experiments — based on the observations from previous experiments.

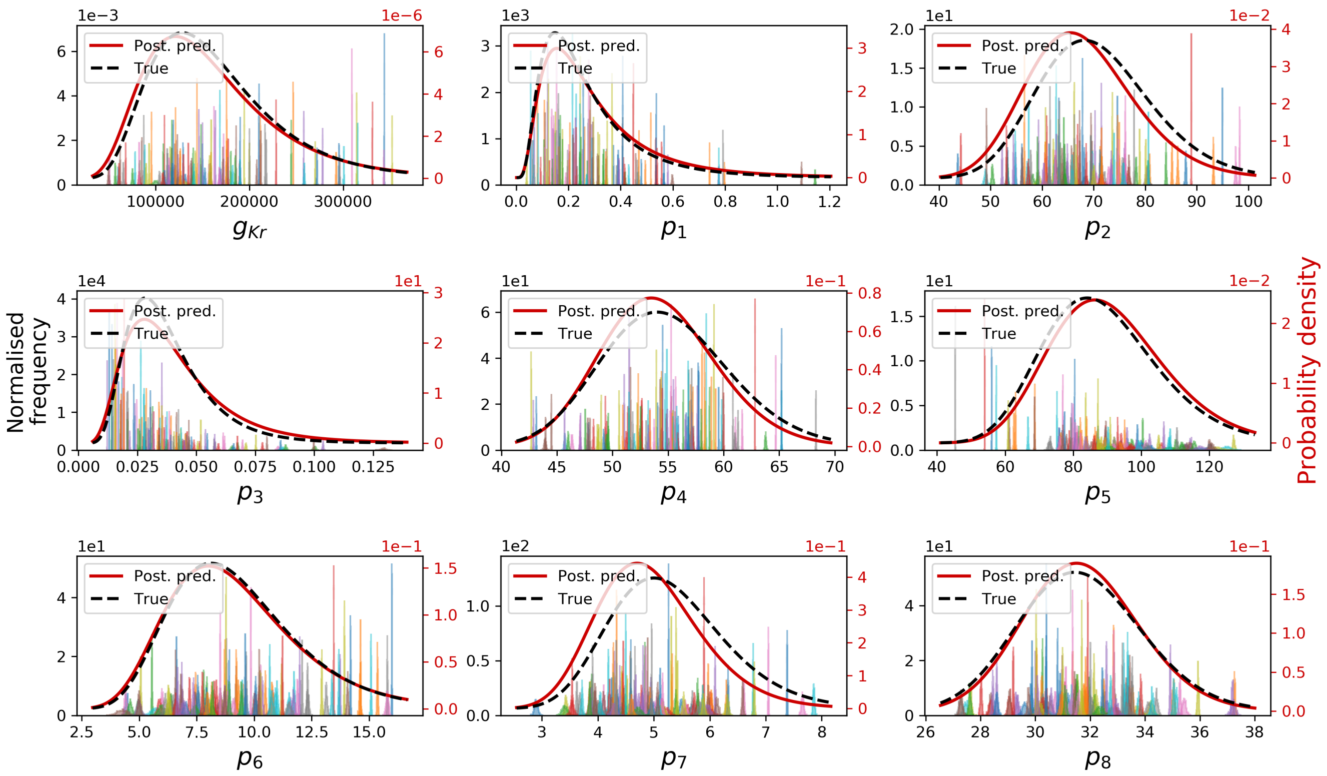

Figure S7. Parameter inference using the hierarchical Bayesian model on synthetic data, with $N_e = 120$. **Left y-axis**: the marginal histograms of the model parameters for each individual experiment. **Right y-axis**: the marginal posterior predictive distributions and the true probability density function that generates the parameters.

We further investigate the correlation between parameters, by trying to recover the correlation matrix corr($\boldsymbol{\theta}$) in Eq. S4. The posterior marginal histograms for each entry of the correlation matrix are shown in Figure S8 (upper triangle). The diagonal is by definition equal to 1, so they are not shown. All inferred marginal posterior distribution for each entry covers the true underlying correlation value (dashed black vertical lines). Therefore it shows us with confidence that our method is suitable for studying the relation *between* model parameters.

Figure S8 (lower triangle) shows the correlation between each pair of parameters. Each contour ring represents the 95% credible intervals of the joint distribution of the two parameters, for both the recovered (blue) and the true (black-dashed) covariance matrices. As long as the main axis of the ellipse is not parallel to the x- or y-axis, it indicates the two parameters are not pairwise-independent. The diagonal shows the sampled predictive posterior distribution before integrated over to give $p(\boldsymbol{\theta}|\cdots)$ shown in Figure S7. Again, it shows that we are able to recover the general shape of the underlying correlation with high accuracy.

In this synthetic study, the correlation matrix that we recovered may not make any physical sense – as we randomly generated it. However, in actual experiments, this correlation matrix tells us which parameters are intrinsically correlated. That is, if there exists any non-zero values, with a good credible interval, in the off-diagonal entries of the recovered correlation matrix, then this informs us how the parameters of model are related.

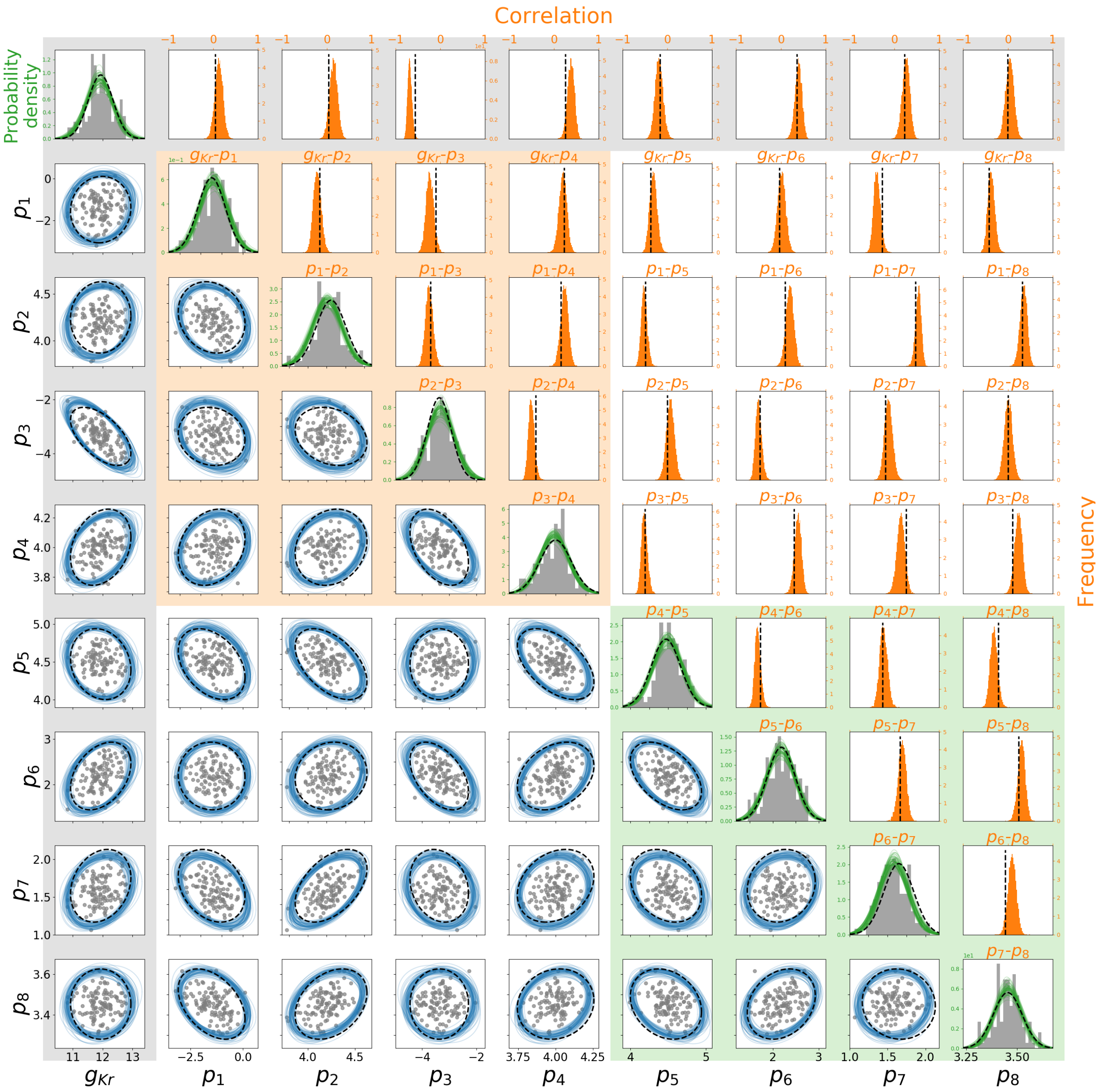

Figure S8. Parameter correlation inference using the hierarchical Bayesian model on synthetic data, with $N_e = 120$. All parameter values shown here are in the natural log-scale. **(Lower-triangle)** Showing the 95% credible region boundary for each pair of parameters reconstructed from the sampled hyperparameters (blue) and the true distribution (black-dashed). **(Diagonal)** Shows the sampled predictive posterior probably density functions before integrated to give $p(\theta|\cdots)$ shown in Figure S7. The marginal probably density functions of the true distribution are shown in dashed black lines for comparison. The parameters of our synthetic data are shown as grey. **(Upper-triangle)** Shows the marginal histograms for each entry of the correlation matrix. The true correlation values are shown as dashed black vertical lines for comparison. The shadings in the background indicate how these parameters relate to the model structure: orange box belongs to the gates a in model, green box gate r, and grey relates to the conductance.

Comparing Pseudo-MwG to MwG All the results above and those in the main text use pseudo-MwG method. Here we provide a brief comparison between our pseudo-MwG method and the MwG for approximating the posterior predictive distribution. We use $N_e = 30$ to demonstrate their similarity. The value was chosen as it is similar to the minimum cell yield we found in the Part II of this paper[6] and it is computationally tractable.

Figure S9 shows the posterior predictive distribution and the histograms of the individual experiments constructed from the pseudo-MwG method (solid lines/filled) and the MwG method (dashed lines/unfilled). It has the same style of plot as in Figure S7, where the left-axes show the marginal histograms and the right-axes show the marginal posterior predictive distributions. The posterior predictive distributions constructed from the pseudo-MwG and MwG look extremely similar. Therefore, with our staircase protocol as the likelihood of the low-level experiments, we are able to simplify our procedure to the pseudo-MwG without losing much accuracy comparing to the MwG algorithm. We expect the agreement to hold for larger N_e as well.

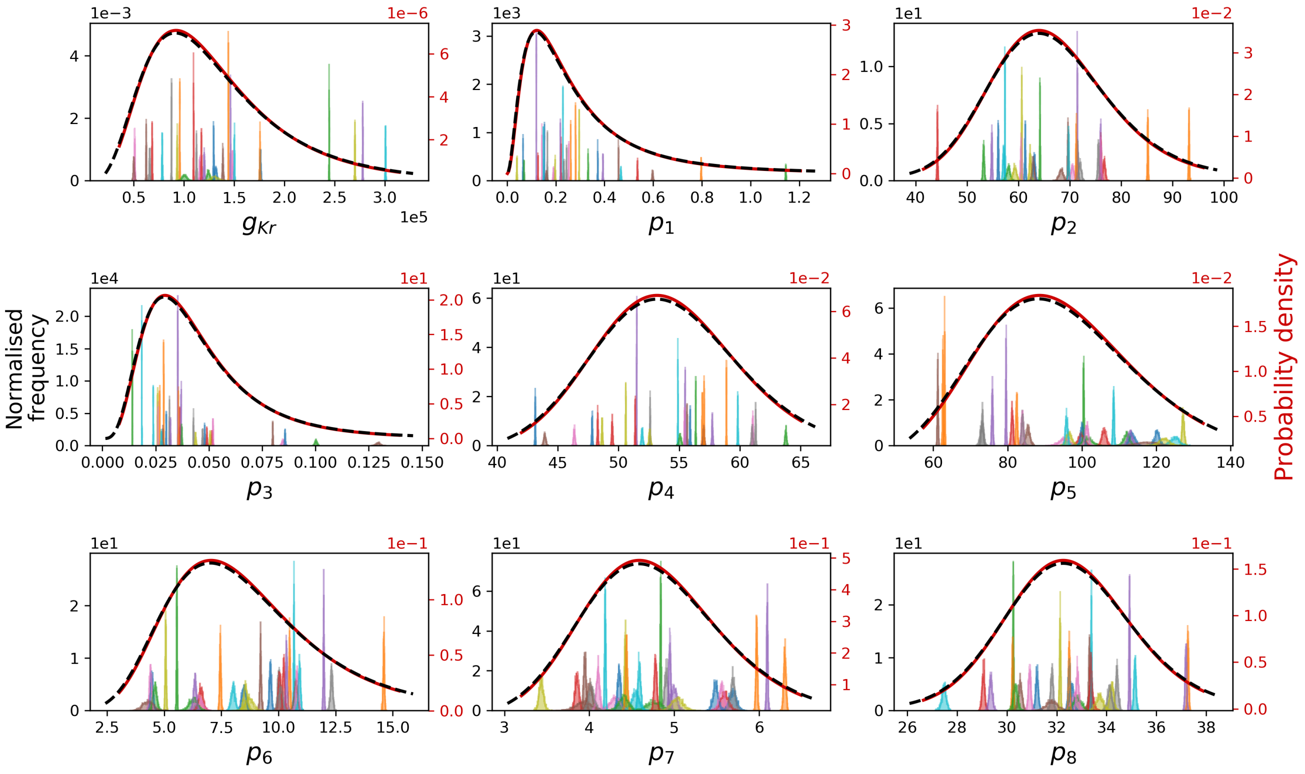

Figure S9. Comparing the hierarchical Bayesian model parameter inference on synthetic data using the pseudo-MwG (solid lines/filled) and the MwG (dashed lines/unfilled) methods, with $N_e = 30$. **Left y-axis**: the marginal histograms of the model parameters for each individual experiment. **Right y-axis**: the marginal posterior predictive distributions.

We also note that we can further simplify our pseudo-MwG, which we shall call it as *simplified pseudo-MwG*, given our information-rich staircase protocol. First we can see that the MCMC distributions, see e.g. Figure S9 marginal histograms, are really narrow relative to spread of each experiment parameters. By approximating these narrow distributions as single points (i.e. delta functions), we can then sample the hyperparameters using Eq. S10–S16, where $\left\{\ln \boldsymbol{\theta}_j\right\}_{j=1}^{N_e}$ become point-estimates of the parameters of the individual experiments j. Figure S10 shows the posterior predictive distribution constructed from the *simplified* pseudo-MwG method (solid lines) and the MwG method (dashed lines). Again, the posterior predictive distributions constructed from the simplified pseudo-MwG and MwG look extremely similar. Therefore, we can further simplify our pseudo-MwG sampling scheme to estimate the full posterior-predictive distribution.

Converging to the true distribution We then check the performance of our method with different numbers of experiments/cells N_e, and confirm that the result converges to the correct answer. We calculate the score with root mean square error (RMSE) for

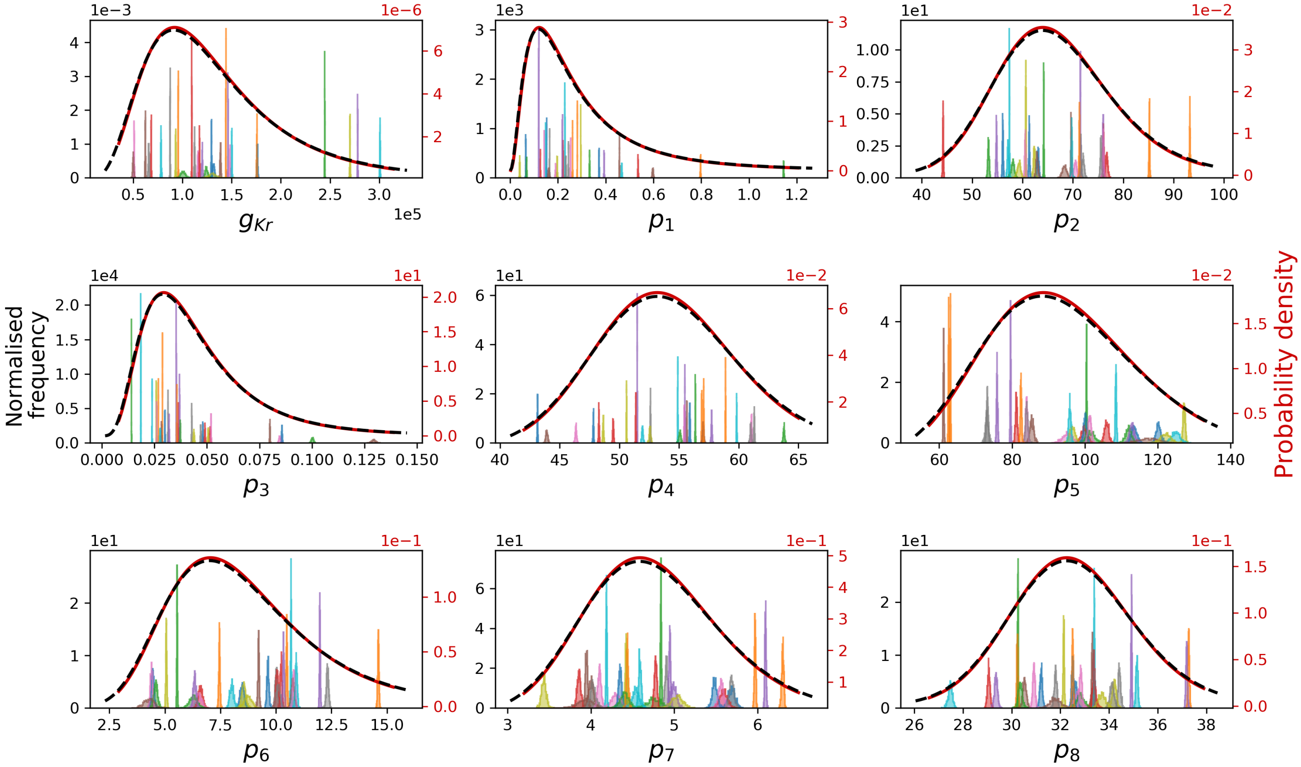

Figure S10. Comparing the hierarchical Bayesian model parameter inference on synthetic data using the *simplified* pseudo-MwG (solid lines) and the MwG (dashed lines) methods, with $N_e = 30$. **Left y-axis**: the marginal histograms of the model parameters for each individual experiment for the MwG method. **Right y-axis**: the marginal posterior predictive distributions.

the correlation matrix, where

$$\text{RMSE of correlation} := \frac{1}{N}\sqrt{\sum_{i}^{N}\sum_{j}^{N}(\text{corr} - \text{corr}_{\text{true}})_{i,j}}, \tag{S18}$$

and its slight variant root mean square percentage error (RMSPE) for standard deviation, where

$$\text{RMSPE of std} := \sqrt{\frac{1}{N}\sum_{i}^{N}\frac{\text{std}^{i} - \text{std}^{i}_{\text{true}}}{\text{std}^{i}_{\text{true}}}}. \tag{S19}$$

We used RMSPE, instead of normal RMSE, for standard deviation to avoid different parameter magnitudes from dominating the calculation.

Figure S11 shows the RMSPE of the standard deviation (left) and RMSE of the correlation (right) as function of the numbers of experiments/cells N_e. For the RMSPE of the standard deviation, Figure S11 (Left), we repeated the above analysis with $N_e = 20, 30, \ldots, 120$ and 125. We can clearly see that the RMSPE of the standard deviation decreases as N_e increases. Hence it is convincing that our method is converging to the true answer in the synthetic data studies.

For the RMSE of the correlation, Figure S11 (Right), we further test the convergence rate of the RMSE value. To run sufficiently large N_e, we simplified our procedure by running only the top-level of the hierarchical Bayesian model, i.e. the *simplified* pseudo-MwG as described above. With this, we ran N_e up to 2×10^4. We plotted both axes in natural-log scale. We then applied a linear regression, in which a slope of -0.516 is obtained. Therefore, we conclude that convergence rate of the RMSE of the correlation is roughly consistent with $\propto 1/\sqrt{N_e}$. We also expect the likely errors in our experiments, with $N_e = 124$, is about 6.4 %, shown as grey lines.

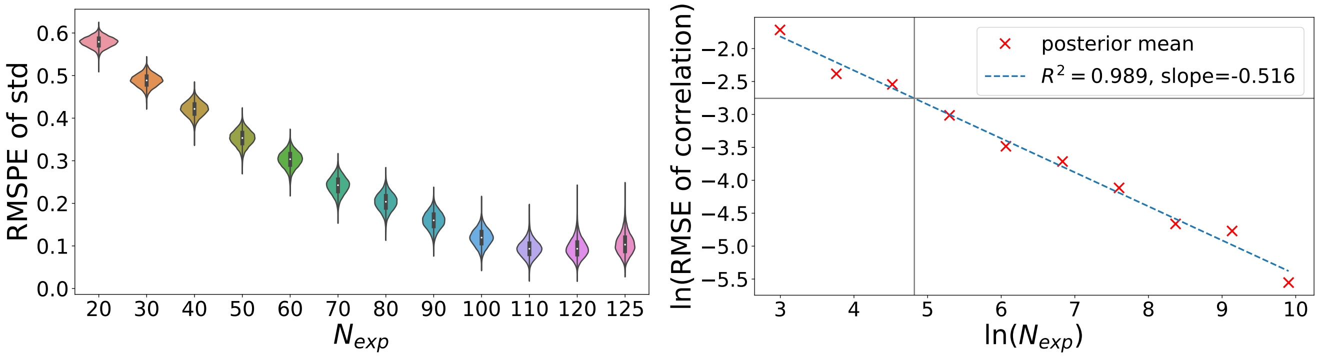

Figure S11. The RMSPE of standard deviation (left) and RMSE of correlation (right) as function of the numbers of experiments/cells N_e. Each violin plot and posterior mean is constructed using 10^4 samples. Grey lines show where $N_e = 124$, with an RMSE value of 0.064.

S7 Sweeps comparison

Here, we check the reproducibility of our results *in the same cells*. We performed the same fitting procedure to the second sweep of our staircase protocol (calibration protocol) recording. First, to assess, if any, intrinsic (or intra-cell) variability[7] in our recordings; and second, to ensure our results are reproducible and biologically meaningful.

Figure S12 shows the fitted parameters comparison between the first sweep (sweep 1) and the second sweep (sweep 2) for all $N = 124$ cells. The line of identity is plotted as grey dashed lines. The two sets of parameters broadly agree, therefore it is convincing that our results are reproducible within the same cells. The intrinsic variability in our recordings are quite small, compared to the extrinsic or experiment-to-experiment variability. Therefore, our analyses focus on the observed experiment-to-experiment variability, and the intrinsic variability are assumed to be negligible.

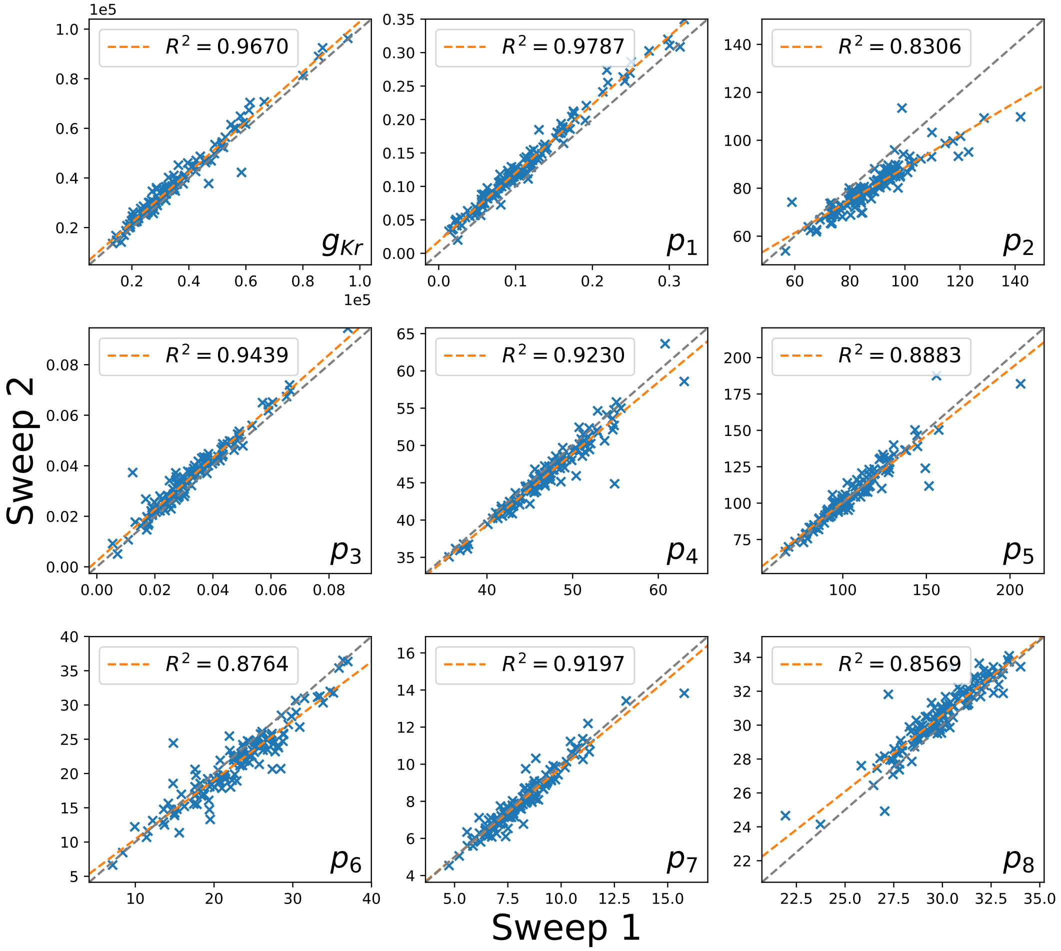

Figure S12. Comparison of the fitted parameters between the first sweep (sweep 1) and the second sweep (sweep 2). Grey dashed lines show the line of identity. The two sets of parameters broadly agree, therefore it is convincing that our results are reproducible within the same cells.

S8 Posterior predictive quantification

We quantify the goodness of the posterior predictive distribution from our hierarchical Bayesian model, compared to the 124 individual experiments, by means of a quantile-quantile (Q-Q) plot and a probability–probability (P-P) plot. The Q-Q (or P-P) plot is a graphical method for comparing two probability distributions, in our case the 124 individual experiments and our posterior predictive distribution $p(\boldsymbol{\theta}|\cdots)$, by plotting their quantiles (or cumulative distributions) against each other.

Note that this is a good test of the LogNormal distribution because we used the pseudo-MwG method, and the individual level parameter fits were not allowed to shift to meet a LogNormal by design as a hierarchical model would generally behave.

Figure S13 and S14 show the Q-Q and P-P plots respectively. In both figures, for each parameter, the marginal posterior predictive distributions are plotted against the posterior mean of the 124 cells. We applied linear regression, shown as orange lines, and they all lie very close to the line of identity (grey dashed lines). These analyses support our results and suggest our posterior predictive distribution, defined by Eq. S17, is a very good description to the distribution of the data.

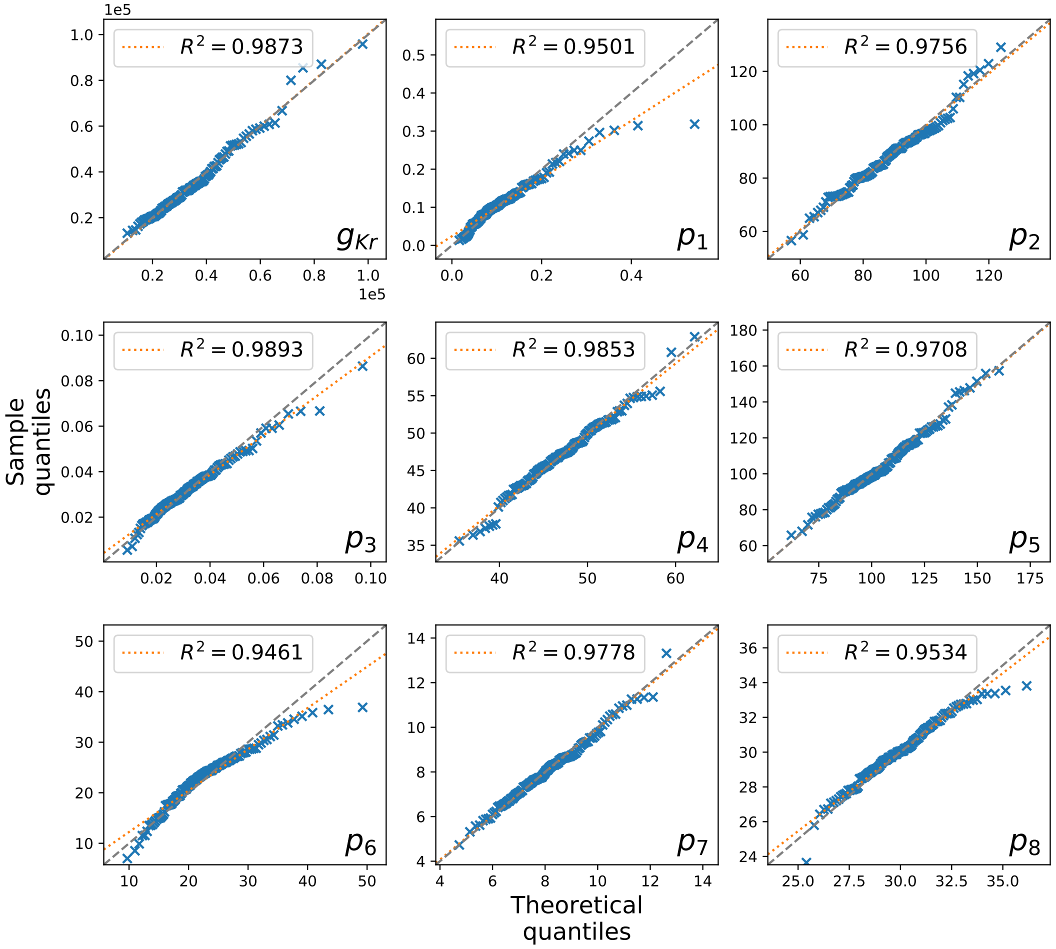

Figure S13. Quantile-quantile (Q-Q) plot of the 124 individual experiments and our posterior predictive distribution. For each parameter, the quantiles of the marginal posterior predictive distribution (theoretical quantiles) are plotted against the quantiles of the posterior mean of the 124 cells (sample quantiles).

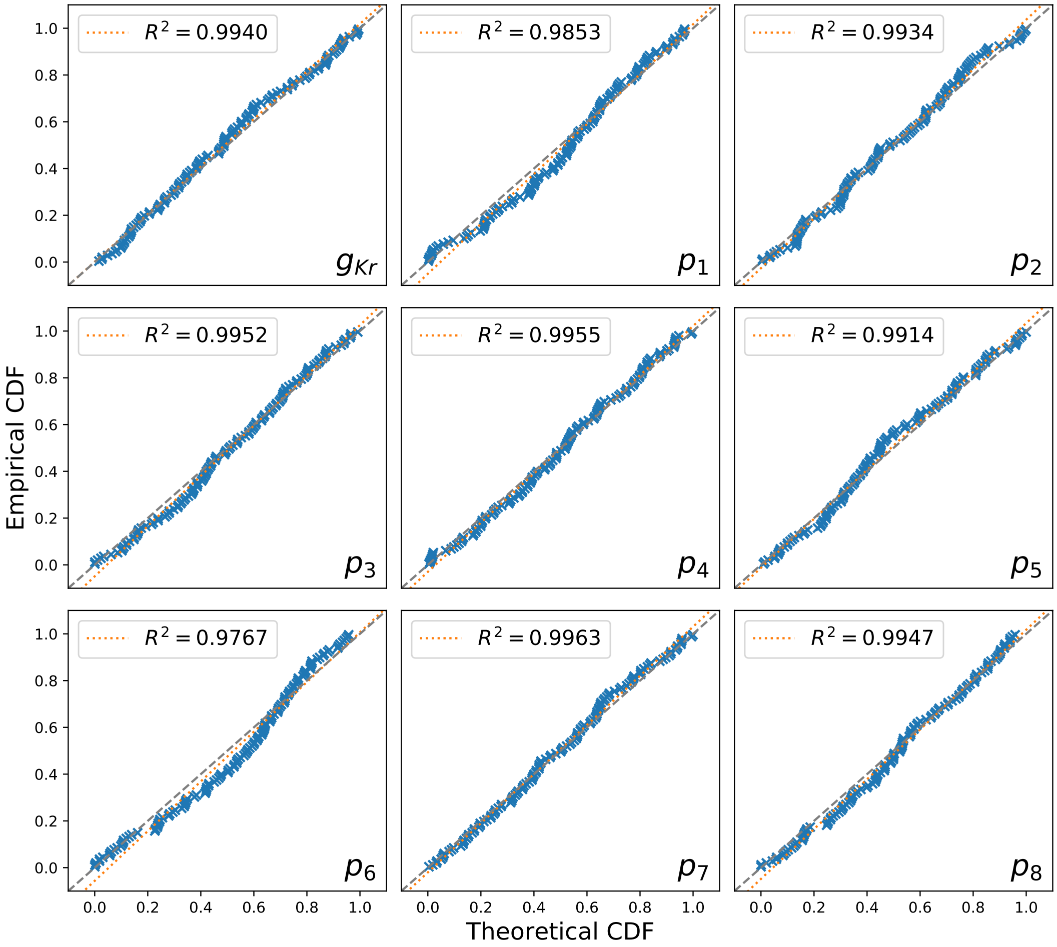

Figure S14. Probability–probability (P-P) plot of the 124 individual experiments and our posterior predictive distribution. For each parameter, the cumulative distribution of the marginal posterior predictive distribution (theoretical CDF) are plotted against the cumulative distribution of the posterior mean of the 124 cells (empirical CDF).

S9 Remaining relative root mean square error (RRMSE) histograms

Here we include the relative root mean square error (RRMSE, given by Eq. 14 in the main text) histograms for the remaining validation protocols 1, 2, and 6 that are not included in the main text due to the space limit. See Figure S15.

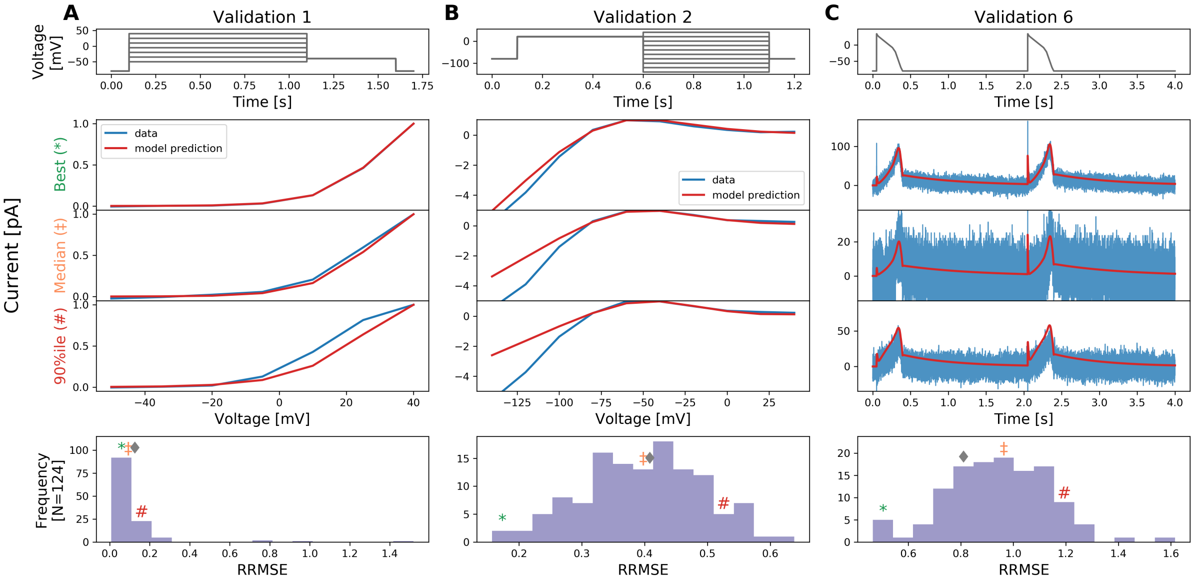

Figure S15. The relative root mean square error (RRMSE, given by Eq. 14 in the main text) histograms for all 124 cells and for validation protocols 1, 2, and 6. Markers indicate the best ($*$), median (‡) and 90th percentile (#) RRMSE values, and diamond marker ♦ indicates the error for the reference traces. For each protocol, the raw traces with the best, median and 90th percentile RRMSE values, for both the model (red) and data (blue) are shown, with the voltage clamp above. Note that the currents are shown on different scales, to reveal the details of the traces.

S10 Practical identifiability of model parameters

In this section we examine the practical identifiability of cell-specific parameters inferred from the experimental measurements. We performed a comparison between two cells (B20 and C17) that had parameters p_1, p_2 at opposite ends of the anti-correlated pairwise plot in Figure 9 in the main text.

Figure S16 shows that all the parameters are tightly constrained within each cell. We observe both the pairwise plots (below the diagonal) and marginal histograms (on the diagonal) from the obtained MCMC chains for the two cells (denoted with purple and brown) within the distributions across cells (denoted with blue and green). Figure S17A shows that these two sets of cell-specific parameters (purple and brown) each have very good cell-specific fits, which do not overlap with fits from a different cell. Indeed the best fits to the data are so tightly constrained within each cell that forward simulations with different samples of the posterior are not distinguishable by eye. Similarly in Figure S17B we see that these cell-specific parameter sets make good cell-specific validation predictions. This is strong evidence that our results are good cell-specific parameter fits, and not overly-narrow distributions that should really overlap.

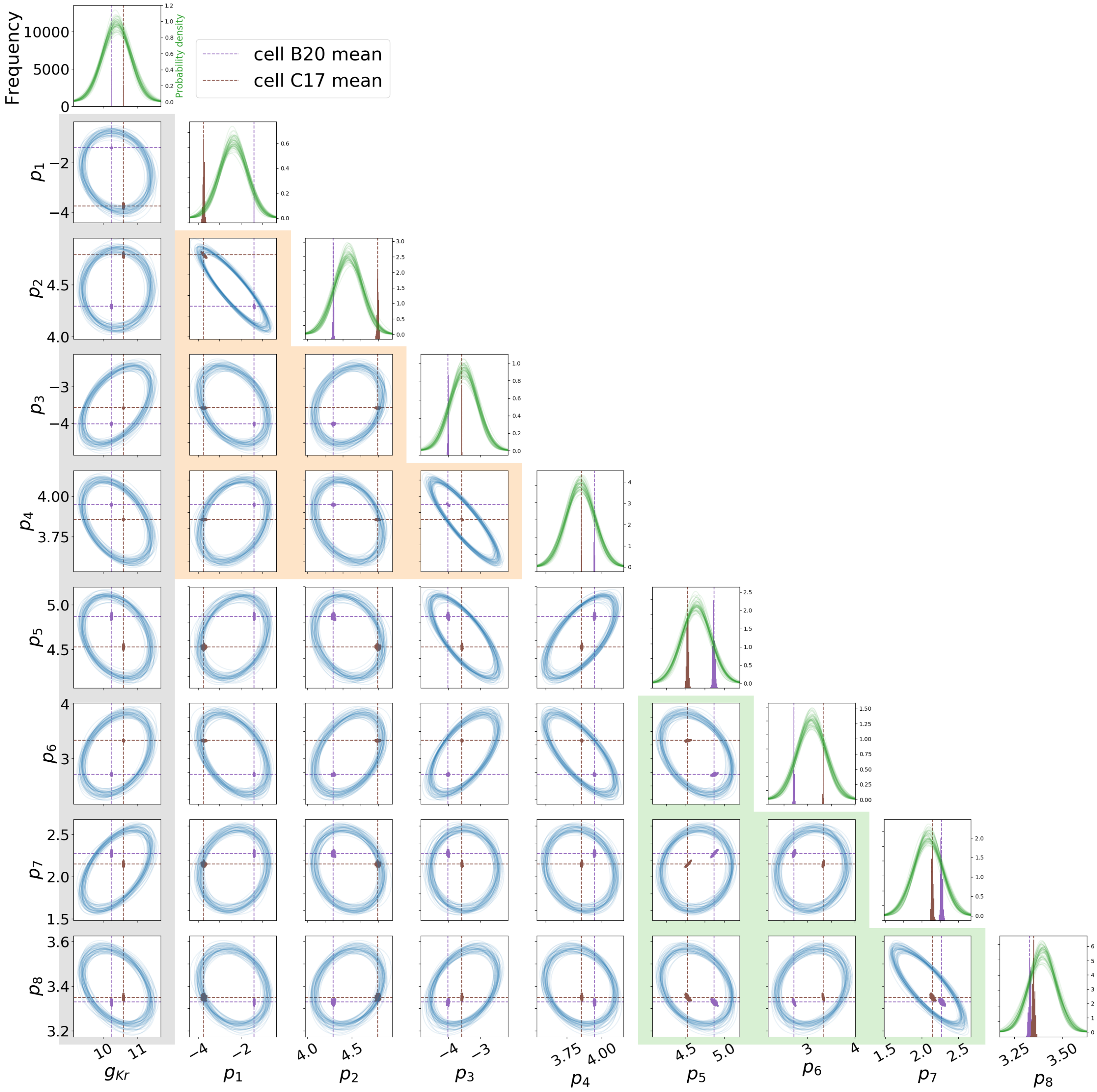

Figure S16. A comparison of the inferred parameters for two example cells: B20 (purple) and C17 (brown). Both the pairwise plots below diagonal and the marginal histograms on the diagonal show samples of the posterior from MCMC chains. Blue ellipses and green distributions (identical to Figure 9 in the main text) are the 95% credible region boundary and posterior probably density functions obtained from the full hierarchical Bayesian model across all cells, capturing the experiment-experiment variability.

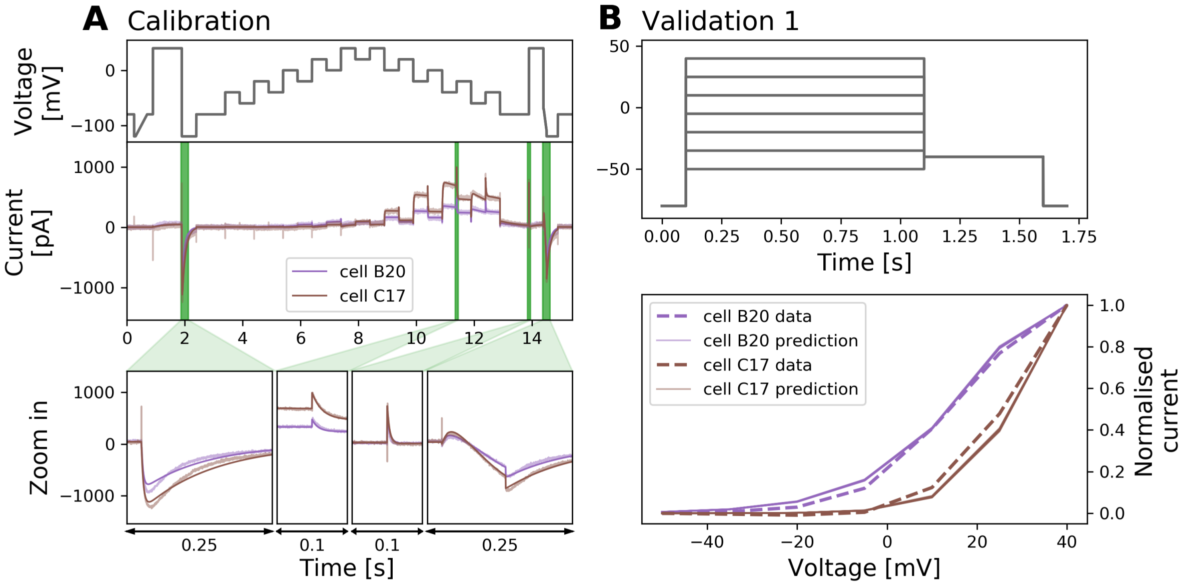

Figure S17. A comparison of cell B20 (purple) and C17 (brown) fits and predictions. **(A)** Shows fits from the posterior (50 samples) from the two cells and corresponding experimental data under the staircase calibration protocol, with corresponding cell-specific data shown in the background. Note that although there are 50 fits plotted, these appear to be a single line for each cell as the parameter samples are so close that the forward simulations are indistinguishable at this scale. **(B)** Predictions based on 50 samples of the cell-specific posteriors under the activation I-V Validation #1 protocol.

S11 Mean model parameters

Table S3 shows the mean values of the model parameters $\boldsymbol{\mu}$ (in Eq. 7 in the main text), which is equivalent to the mean of the posterior predictive distribution, and the two set of 95% credible intervals. '95th %ile (mean)' is the 95% credible intervals for the uncertainty of the top-level mean parameter vector $\boldsymbol{\mu}$, which we describe it as the representative of our uncertainty in the underlying physiology (see main text Discussion). '95th %ile (exp)' is the 95% credible intervals of the full posterior predictive distribution, which represents the variability of the experiments.

Table S4 compare the mean values of the steady state activation and inactivation parameters of our model against the reported values in the literature[8]; Figure S18 shows the respective predictions.

	g_{Kr} $[pS]$	p_1 $[s^{-1}]$	p_2 $[V^{-1}]$	p_3 $[s^{-1}]$	p_4 $[V^{-1}]$	p_5 $[s^{-1}]$	p_6 $[V^{-1}]$	p_7 $[s^{-1}]$	p_8 $[V^{-1}]$
mean	3.23e+4	9.48e-2	8.69e+1	2.98e-2	4.69e+1	1.04e+2	2.19e+1	8.05e+0	2.99e+1
95th %ile (mean)	3.e+4<br>3.48e+4	8.43e-2<br>1.06e-1	8.45e+1<br>8.93e+1	2.76e-2<br>3.23e-2	4.60e+1<br>4.78e+1	1.00e+2<br>1.07e+2	2.08e+1<br>2.31e+1	7.77e+0<br>8.34e+0	2.95e+1<br>3.03e+1
95th %ile (exp)	1.42e+4<br>7.36e+4	2.59e-2<br>3.46e-1	6.36e+1<br>1.19e+2	1.24e-2<br>7.18e-2	3.80e+1<br>5.78e+1	7.06e+1<br>1.52e+2	1.2e+1<br>4.01e+1	5.43e+0<br>1.19e+1	2.59e+1<br>3.45e+1

Table S3. The mean values of the model parameters model parameters $\boldsymbol{\mu}$ (in Eq. 7 in the main text). The two set of 95th percentiles are the 95% credible intervals of (mean) the uncertainty of the mean parameter vector $\boldsymbol{\mu}$; and (exp) the full posterior predictive distribution.

	activation $V_{1/2}$ [mV]	activation k [mV]	inactivation $V_{1/2}$ [mV]	inactivation k [mV]
Our mean (n=124)	-8.6	7.5	-49.3	-19.3
Sanguinetti et al.[8] (n=10)	-15.0	7.9	-49.0	-28.0

Table S4. A comparison of the mean values of the steady state activation and inactivation parameters of our model against the reported values in Sanguinetti et al. 1995[8].

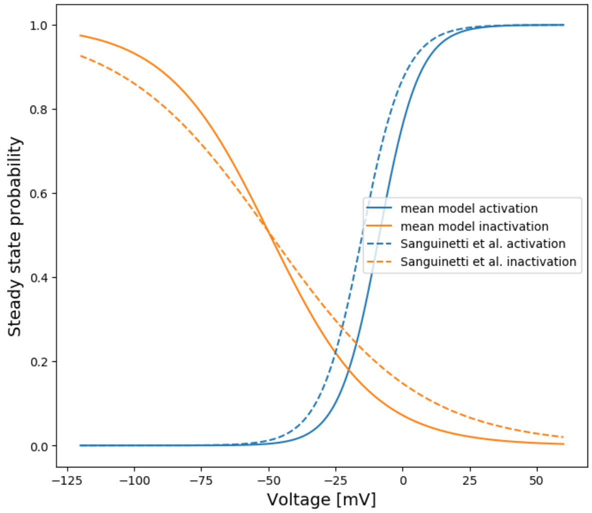

Figure S18. A comparison of the mean value parameter predictions of the steady state activation and inactivation between our model and Sanguinetti et al. 1995[8].

S12 Estimated voltage error and other quality control parameters

To investigate the possibility of all the quality control parameters having bearing on the estimated voltage error, we plot scatter plots of the estimated voltage error ΔV_j (see main text Discussion) against R_{seal}, C_{m}, R_{series}, g_{leak}, and E_{leak}, as shown in Figure S19. However, no obvious correlation between these values is observed.

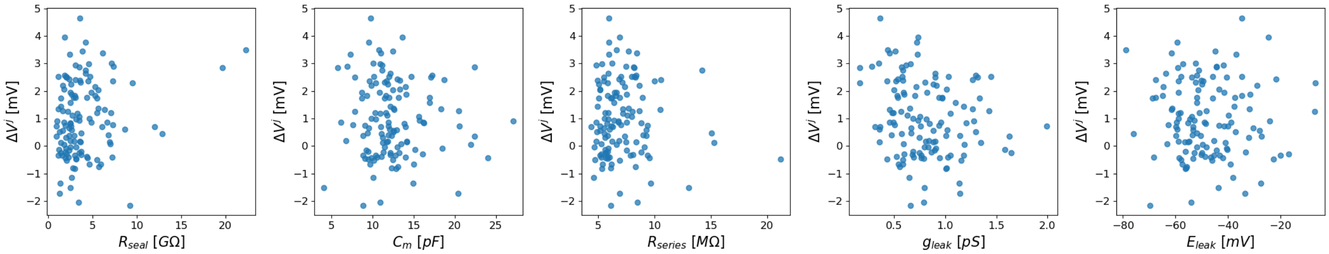

Figure S19. Scatter plots of the voltage error ΔV_j (see main text Discussion) against R_{seal}, C_{m}, R_{series}, g_{leak}, and E_{leak}.

S13 Estimated voltage error and parameter variability

Figure S20 shows an extended version of Figure 9 (Lower triangle) in the main text. Each individual well's parameter set (originally grey dots) is colour-coded in terms of the ordering of the estimated voltage error ΔV_j values, with cyan representing the wells with the lowest ΔV_j values and navy representing the largest ΔV_j. The trend in the parameter values as estimated voltage error ΔV_j increases qualitatively agrees with the directions of red lines indicating the predicted effect of ΔV_j in parameters (see Discussion in the main text). This provides further evidence of the hypothesis in the Discussion in the main text that varying patch clamp artefacts are a leading cause of variability in parameter sets across wells.

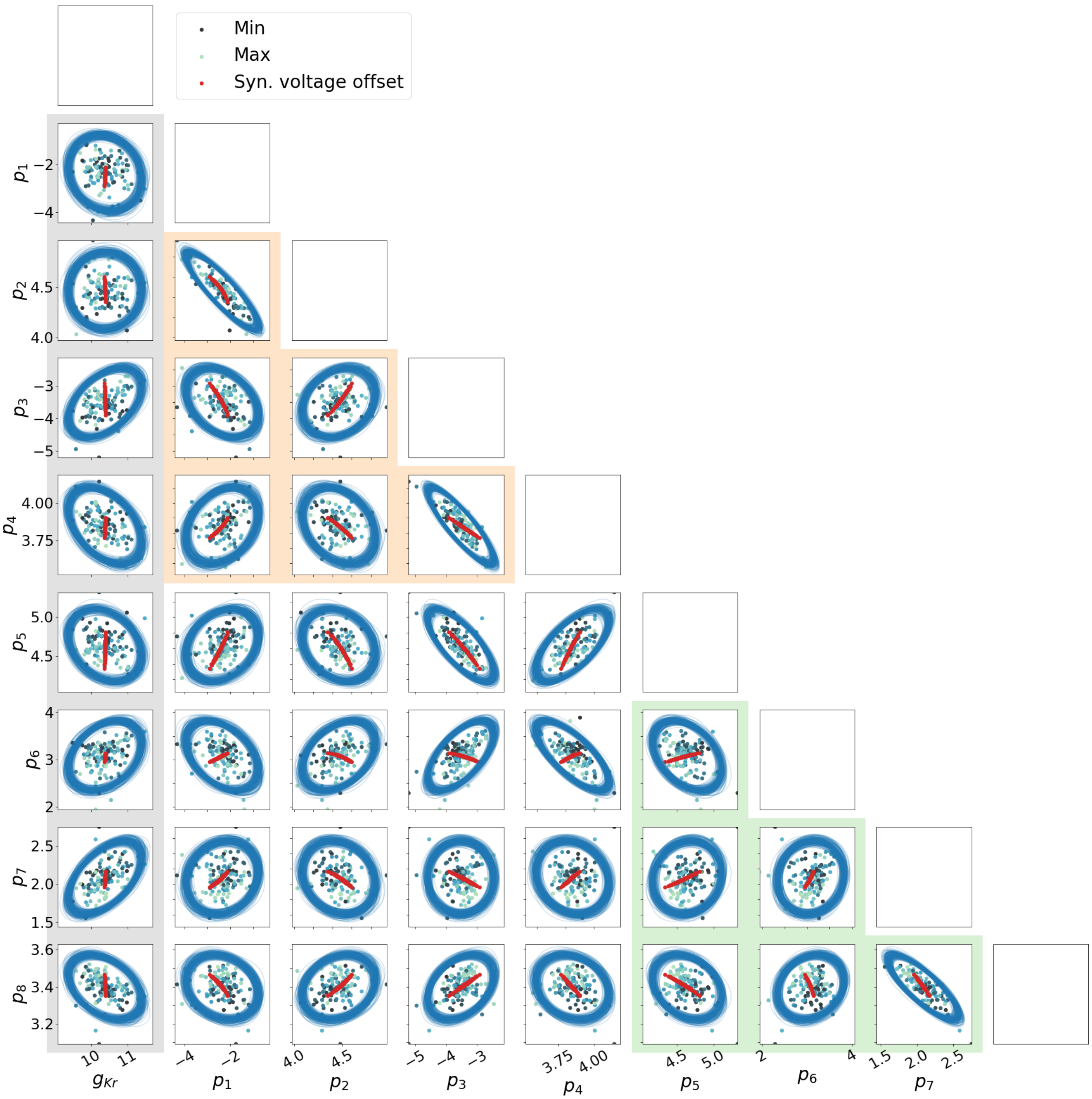

Figure S20. Extension of Figure 9 in the main text. Each individual parameters (originally grey dots) is colour-coded in terms of the ordering of the estimated voltage error ΔV_j (see main text Discussion) values, with cyan representing the wells with the lowest ΔV_j values and navy representing the largest ΔV_j.

References

1. Beattie, K. A. *et al.* Sinusoidal voltage protocols for rapid characterisation of ion channel kinetics. *The J. Physiol.* **596**, 1813–1828 (2018). DOI 10.1113/JP275733.
2. Hansen, N. *The CMA Evolution Strategy: A Comparing Review*, 75–102 (Springer Berlin Heidelberg, Berlin, Heidelberg, 2006).
3. Jasra, A., Stephens, D. A. & Holmes, C. C. On population-based simulation for static inference. *Stat. Comput.* **17**, 263–279 (2007).
4. Haario, H., Saksman, E., Tamminen, J. *et al.* An adaptive metropolis algorithm. *Bernoulli* **7**, 223–242 (2001).
5. Gilks, W. R., Best, N. & Tan, K. Adaptive rejection metropolis sampling within gibbs sampling. *Appl. Stat.* 455–472 (1995).
6. Lei, C. L. *et al.* Rapid characterisation of hERG potassium channel kinetics II: temperature dependence. *Biophys. J.* **this issue** (2019).
7. Mirams, G. R., Pathmanathan, P., Gray, R. A., Challenor, P. & Clayton, R. H. Uncertainty and variability in computational and mathematical models of cardiac physiology. *The J. Physiol.* **594**, 6833–6847 (2016).
8. Sanguinetti, M. C., Jiang, C., Curran, M. E. & Keating, M. T. A mechanistic link between an inherited and an acquird cardiac arrthytmia: Herg encodes the ikr potassium channel. *Cell* **81**, 299–307 (1995).
